# Comprehensive Analysis of Disease Pathology in Immunocompetent and Immunocompromised Hosts following Pulmonary SARS-CoV-2 Infection

**DOI:** 10.3390/biomedicines10061343

**Published:** 2022-06-07

**Authors:** Santhamani Ramasamy, Afsal Kolloli, Ranjeet Kumar, Seema Husain, Patricia Soteropoulos, Theresa L. Chang, Selvakumar Subbian

**Affiliations:** 1 Public Health Research Institute, Rutgers-New Jersey Medical School, Newark, NJ 07103, USA; santhuvet@gmail.com (S.R.); ak1482@njms.rutgers.edu (A.K.); rk879@njms.rutgers.edu (R.K.); changth@njms.rutgers.edu (T.L.C.); 2 The Genomics Center at Rutgers-New Jersey Medical School, Newark, NJ 07103, USA; husainse@njms.rutgers.edu (S.H.); soteropa@njms.rutgers.edu (P.S.)

**Keywords:** immune suppression, COVID-19, immunopathology, extrapulmonary, antibody, RNAseq, gene networks, thrombosis, animal models

## Abstract

The Coronavirus disease 2019 (COVID-19) pandemic disproportionately affects immunocompetent and immunocompromised individuals, with the latter group being more vulnerable to severe disease and death. However, the differential pathogenesis of SARS-CoV-2 in the context of a specific immunological niche remains unknown. Similarly, systematic analysis of disease pathology in various extrapulmonary organs in immunocompetent and immunocompromised hosts during SARS-CoV-2 infection is not fully understood. We used a hamster model of SARS-CoV-2 infection, which recapitulates the pathophysiology of patients with mild-to-moderate COVID-19, to determine the dynamics of SARS-CoV-2 replication and histopathology at organ-level niches and map how COVID-19 symptoms vary in different immune contexts. Hamsters were intranasally infected with low (LD) or high (HD) inoculums of SARS-CoV-2, and the kinetics of disease pathology and viral load in multiple organs, antibody response, inflammatory cytokine expression, and genome-wide lung transcriptome by RNAseq analysis were determined and compared against corresponding responses from chemically induced immunocompromised hamsters. We observed transient body weight loss proportional to the SARS-CoV-2 infectious dose in immunocompetent hamsters. The kinetics of viral replication and peak viral loads were similar between LD and HD groups, although the latter developed more severe disease pathology in organs. Both groups generated a robust serum antibody response. In contrast, infected immunocompromised animals showed more prolonged body weight loss and mounted an inadequate SARS-CoV-2-neutralizing antibody response. The live virus was detected in the pulmonary and extrapulmonary organs for extended periods. These hamsters also had persistent inflammation with severe bronchiolar-alveolar hyperplasia/metaplasia. Consistent with the differential disease presentation, distinct changes in inflammation and immune cell response pathways and network gene expression were seen in the lungs of SARS-CoV-2-infected immunocompetent and immunocompromised animals.

## 1. Introduction

Coronavirus disease 2019 (COVID-19), caused by the severe acute respiratory syndrome coronavirus-2 (SARS-CoV-2), continues to be a significant global health concern. To date, at least 250 million cases and 5 million deaths have been confirmed worldwide [1]. COVID-19 pathology is associated with host immune alterations, which begin during the early stages of SARS-CoV-2 infection. This results in hyper-inflammation that progresses to acute respiratory distress syndrome (ARDS) and ultimately death in severe cases [2,3]. COVID-19 patients with delayed virus clearance (>20 days) were at higher risk of progression to ARDS and death [4,5]. Pre-existing host immunosuppression is a significant risk factor for delayed virus clearance and compromised antibody response against SARS-CoV-2 [6]. However, the intricate association between immunosuppression and its effect on the progression of SARS-CoV-2 infection remains unclear.

In addition to pulmonary pathology, including pneumonia and ARDS, the severity of COVID-19 can manifest as a multisystem disorder that can compromise neuronal, cardiovascular, gastrointestinal, urogenital, endocrine, and other systems [7]. Therefore, it is imperative to analyze the extent of pathological complications caused by SARS-CoV-2 in the extrapulmonary system, which are currently poorly understood [8]. However, a key challenge to characterizing disease pathology caused by SARS-CoV-2 in various tissues is the biosafety-based restrictions on autopsies of patients who died of COVID-19 [9]. To alleviate this limitation, animal models of SARS-CoV-2 have been utilized [10,11,12]. These studies have contributed to our understanding of the host–pathogen interactions during SARS-CoV-2 infection. However, a majority of the reports on COVID-19 pathology in animal models are confined mainly to the respiratory tract, and the systematic, longitudinal analysis of extrapulmonary pathology is scant [10,11,12].

The extent of protection against progressive SARS-CoV-2 infection into severe COVID-19 correlates with the viral-neutralizing antibody titer in patient sera [13]. Thus, the antibody-mediated immune response, elicited in the host upon vaccination and/or exposure to SARS-CoV-2, has a host-protective role against disease progression. However, it has been shown that immunocompromised individuals, such as those with cancer or HIV-1 infection, lack a strong antibody response against SARS-CoV-2 upon infection, which would explain the occurrence of severe COVID-19 in these individuals [6]. In addition, the effect of reducing hyper-inflammation by treatment with steroids and other immunosuppressive drugs, such as those used to treat cancer, on the progression of SARS-CoV-2 infection, and associated COVID-19 pathology, are not fully understood. Since immunocompromised hosts are more vulnerable to severe COVID-19, it is crucial to understand the impact of immunosuppression caused by immunosuppressive therapy on the antibody response and, subsequently, on the progression of SARS-CoV-2 infection. A comprehensive and comparative analysis of the kinetics of viral replication and disease pathology during SARS-CoV-2 infection in various organs of an immunocompetent and immunocompromised host would help to devise better disease control strategies against COVID-19.

The current study reports a systematic and comprehensive analysis of disease severity in various organs of a Golden Syrian hamster model of pulmonary SARS-CoV-2 infection. We analyzed the kinetics of viral replication, antibody response, cytokine storm marker expression, and disease pathology in pulmonary and extrapulmonary tissues of immunocompetent and immunocompromised hamsters infected intranasally with a low or high dose of a virulent strain of SARS-CoV-2. Further, using genome-wide RNAseq analysis of the lungs, we determined the differential immune correlates of disease pathogenesis between immunocompetent and immunocompromised hamsters during SARS-CoV-2 infection. Our data suggest that the disease pathology in the pulmonary and extrapulmonary systems is the key and prominent indicator for the severity of progressive SARS-CoV-2 infection over the serum antibody response and viral load in internal organs. The differential expression of network and pathway genes between immunocompetent and immunocompromised hamsters corroborates the underpinning differential SARS-CoV-2 pathogenesis in these animals.

## 2. Materials and Methods

### 2.1. Virus and Cell Lines

SARS-CoV-2 (strain USA-WA1/2020)-infected Vero E6 cell supernatant was obtained from BEI Resources (BEI Resources, Manassas, VA, USA). Virus propagation, virus titration, infectivity assays, and antibody titration were performed using Vero E6 cells (ATCC, Manassas, MA, USA) [14,15]. Experiments involving infectious SARS-CoV-2 were conducted in Biosafety level 3 facilities at Rutgers University, as per approved standard operating procedures. Unless specified, all chemicals and reagents were purchased from Sigma-Aldrich (Sigma-Aldrich, St. Louis, MO, USA).

### 2.2. SARS-CoV-2 Infectivity Titration

We performed a plaque assay to determine live viruses in the inoculum and tissue homogenates, as described previously [16]. The Vero E6 cell monolayer was infected with SARS-CoV-2 using DMEM media (supplemented with 2% FBS). The plaques were visualized on the 3rd day by staining with 0.2% crystal violet, and plaque-forming units per mL of inoculum were determined [16].

### 2.3. Golden Syrian Hamster Infection and Sample Collection

Seventy-five (*n* = 75) male Golden Syrian hamsters (*Mesocricetus auratus*) between 6 and 8 weeks old were purchased (Envigo corporation, Denver, PA, USA) and housed at two animals/cage. Feed and water were given ad libitum throughout the experiment. The animals were acclimatized for seven days in the BSL3 facilities. Body weight, food, and water intake were monitored twice a day for each animal throughout the experiment. 

Immunocompetent hamsters*:* For intranasal infection, SARS-CoV-2 was prepared in two doses; low dose (LD; 10^2.5^ PFU) and high dose (HD;10^6^ PFU) in 50 µL of sterile 1xPBS. LD was administered to 30 animals, and HD was inoculated to 15 healthy hamsters. Due to a very low viral load, the LD-inoculated animals might show variability in the amount of virus lodged into the lungs; therefore, we included 6 animals per timepoint in this group to account for this variability. We used only male animals due to their ease of handling and ability to house together. Since we used only one biological sex (male) and observed more consistent viral delivery to the lungs, we used 3 animals from the HD group to get enough statistical values for testing. For the uninfected control group, 5 hamsters were intranasally inoculated with 50 µL of sterile 1xPBS. 

Immunosuppression treatment of hamsters: Cyclophosphamide was injected intra-peritoneally into 30 animals at 70 mg/kg on the day of intranasal SARS-CoV-2 inoculation with 10^2.5^ PFU (CP-LD), and then every 3 days until the end of the experimental time point (16 dpi). This method has previously been shown to immunocompromise hamsters and causes them to be more vulnerable to progressive SARS-CoV infection [17]. Five animals treated with Cyclophosphamide, as mentioned above, were intranasally inoculated with 50 µL of sterile 1xPBS as the control group. Six animals from the LD and Cyclophosphamide-LD (*n* = 6) groups and three animals from the HD group were euthanized 2, 4, 7, 12, and 16 days post-infection. Blood was collected by cardiac puncture before necropsy. The turbinates, larynx and trachea, lung, heart, liver, spleen, adrenal, kidney, colon, epididymal fat, brain, and eyes were collected and weighed aseptically. A portion of the harvested tissues was used for the homogenization for plaque assay, stored in 10% buffered formalin for histopathology analysis, or stored in Trizol (ThermoFisher Scientific, Waltham, MA, USA) at −80 °C for RNA extraction.

### 2.4. Histopathology Analysis

The tissues were fixed in 10% buffered formalin, made into paraffin blocks, sectioned to 5-micron thickness, and stained with hematoxylin and eosin or Trichrome, as described previously [18]. The identity of samples was blinded before analysis, and histopathological examination was performed by a board-certified veterinary virologist (S.R) using the EVOS FL Cell imaging system (ThermoFischer Scientific, Waltham, MA USA). Histopathology images were organized and labeled using Adobe Photoshop v22.1.1 and Adobe Illustrator v25.1. Spleen white pulp cells were quantified manually using ImageJ (NIH, Bethesda, MD, USA). The pulmonary pathology was scored (0–4/5) based on the degree of mononuclear infiltration, edema, alveolar/bronchiolar hyperplasia, emphysema, vascular lesions, bronchiolar/arteriolar smooth muscle thickening, and foamy macrophages.

### 2.5. Immunohistochemistry Analysis

The formalin-fixed, paraffin-embedded tissue blocks were sliced into 5-micron sections and processed following standard procedures [18]. The sections were stained with rabbit anti-hamster ACE2 antibody (Product no. HPA000288, Millipore Sigma, Burlington, MA, USA) followed by Alexa-488 labeled anti-rabbit secondary antibodies (Cat no. ab150077, Abcam, MA, USA), as described previously [19]. For the mRNA-FISH technique, labeled smRNA-FISH probes (Biosearch Technologies, Dexter, MI, USA) corresponding to specific host genes were added to the top of the sections and processed as described previously [19]. Images were captured using Axiovert 200 M inverted fluorescence microscope (Zeiss, Oberkochen, Germany) using 20× or 63× oil-immersion objective with Prime sCMOS camera (Photometrics, Tucson, AZ, USA) by Metamorph image acquisition software (Molecular Devices, San Jose, CA, USA), ImageJ software was used for analyses. The number of cells positive for a marker was normalized to the total number of cells in each field (3–5 fields containing at least 100 cells were analyzed per sample). Further, GraphPad Prism-8 (GraphPad Software, San Diego, CA, USA) was used for the statistical analysis of data. *p*-values < 0.05 were considered statistically significant.

### 2.6. Virus Infectivity Assays on Tissues

Tissue homogenates were centrifuged, and the supernatant was filtered through a 0.45 µ filter. The filtrate was diluted in serum-free DMEM, and 400 µL was used to infect the Vero E6 cell monolayers in the 6-well plates and plaque assays were performed as described previously [20]. The plaque-forming units per gram of tissues were determined.

### 2.7. Plaque Reduction Neutralization (PRNT) Assay

Hamster serum samples were heat-inactivated at 56 °C for 30 min and diluted in DMEM (1:16, 1:32, 1:80, 1:160, 1:320, 1:640, 1:1280). Each dilution was incubated with 20–30 PFU of SARS-CoV-2 at 37 °C for 1 h. The virus-antibody complexes were added to Vero E6 cells in a 6-well plate at 37 °C for 1 h. The PFU assay was performed as described above. The PRNT_90_ was calculated as the reciprocal of serum dilution, which inhibited the number of plaques by 90% compared to virus-DMEM control without antibodies [20].

### 2.8. RNA Isolation from Hamster Lungs

Total RNA was extracted from the lungs of uninfected and SARS-CoV-2-infected hamsters with or without immune suppression using Trizol reagent and purified by RNeasy mini columns (Qiagen, CA, USA), as described previously [21]. cDNA synthesis was performed using a High-Capacity cDNA Reverse Transcription Kit with 500 ng of total RNA, as per the standard protocol (Applied Biosystems, Waltham, MA, USA).

### 2.9. RNAseq Analysis of Lung Transcriptome

The quality of RNA was checked for integrity on an Agilent 2200 TapeStation (Agilent Technologies, CA, USA), and samples with RNA integrity number (RIN) > 7.0 were used for subsequent processing. Total RNA was subjected to two rounds of poly(A) selection using oligo-d(T)25 magnetic beads (New England Biolabs, CA, USA). The Illumina-compatible RNAseq library was prepared using NEB next ultra RNAseq library preparation kit. The cDNA libraries were purified using AmpureXP beads and quantified on an Agilent TapeStation and Qubit 4 Fluorometer (ThermoFisher Scientific, Waltham, MA, USA). An equimolar amount of barcoded libraries were pooled and sequenced on the Illumina NovaSeq platform (Illumina, San Diego, CA, USA) using the 1 × 100 cycles configuration of the CLC Genomics Workbench 20.0.4 version (Qiagen, Valencia, CA, USA). De-multiplexed fastq files from RNA-Seq libraries were imported into the CLC software. Bases with low quality were trimmed, and reads were mapped to reference genome *Mesocricetus auratus* (assembly BCM_Maur_2.0). The aligned reads were obtained using the RNA-Seq Analysis Tool of the CLC Genomics Workbench. Statistical analysis of differentially expressed genes was carried out based on a negative binomial model using the CLC Genomic Workbench tool. Replicates were averaged and statistically differentially expressed genes (SDEG) were identified with a false discovery rate (FDR) *p*-value < 0.05 and fold change of an absolute value > 1.5. We compared the following groups: (1) immunocompetent uninfected versus SARS-CoV-2-infected animals at 4 and 16 dpi; (2) immunosuppressed SARS-CoV-2-infected animals at 4 and 16 dpi versus immunocompetent animals at respective time points. To account for the gene expression changes caused by immunosuppression treatment, the data from the immunosuppressed/infected animals were normalized to the corresponding uninfected (immunosuppressed) controls before comparison analysis with the corresponding immunocompetent counterparts.

### 2.10. Gene Network and Pathway Analysis

The SDEG was subjected to further visualization analysis, including a heat map using Partek Genomics Suite version 7.0 (Partek Inc., St. Louis, MO, USA) software, as described previously [21,22]. The SDEG were also analyzed by using Ingenuity Pathway Analysis (IPA) software (Ingenuity^®^ Systems, Inc., Redwood City, CA, USA) to determine the networks and pathways that are affected in SARS-CoV-2-infected and/or uninfected hamster lungs with or without immunosuppression at 4 and 16 dpi, as described previously [21,22]. In this software, the significance of a network or pathway is determined by both the *p*-value calculated using the right-tailed Fisher’s Exact Test and the Z-score. For these statistical calculations, the total number of genes in the IPA knowledgebase was compared and computed against the experimental data set from each test group.

### 2.11. Determination of Total Viral Load by Quantitative PCR

Quantitative PCR was performed using total RNA and SARS-CoV-2 N gene-specific primers (SARS-CoV-2_N-F1: GTGATGCTGCTCTTGCTTTG and SARS-CoV-2_N-R1: GTGACAGTTTGGCCTTGTTG) and Power SYBR Green PCR MasterMix as per the manufacturer’s protocol (Applied Biosystems, Waltham, MA, USA) [23]. The purified N gene PCR products were used to prepare a standard curve and determine the viral copy numbers in the lung samples. The list of primers used in this study is available in Table S5.

### 2.12. Statistical Analysis

Data from immunocompetent and immunocompromised SARS-CoV-2-infected hamsters were presented relative to corresponding uninfected (control) groups. Statistical analysis was performed using GraphPad Prism-8 (GraphPad Software, La Jolla, CA, USA), and the mean ± standard deviation (SD) values were plotted as graphs. Unpaired Student’s *t*-test with Welch correction was used to analyze the data between two groups, and one-way ANOVA with Tukey’s correction was used for multiple group comparison. For all the experimental data, *p* ≤ 0.05 was considered statistically significant.

### 2.13. Ethics Statement

All animal procedures were performed in bio-safety level 3 (BSL3) facilities according to the ethical policies and procedures approved by the Rutgers University Institutional Animal Care and Use Committee (IACUC Approval no. PROTO202000103), which is consistent with the policies of the American Veterinary Medical Association (AVMA), the United States Center for Disease Control (CDC), and the United States Department of Agriculture (USDA).

## 3. Results

### 3.1. Distinct Disease Progression in SARS-CoV-2-Infected Immunocompetent and Immunocompromised Hamsters

**Figure 1:**
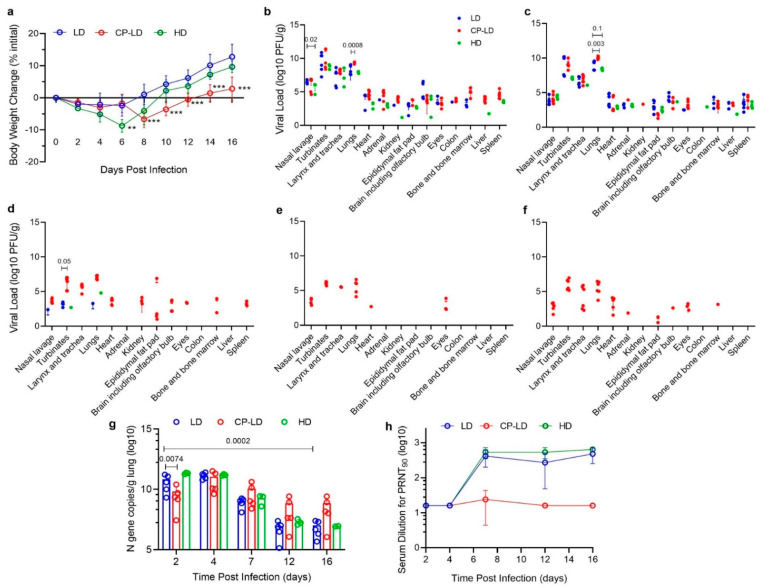
COVID-19 disease in SARS-CoV-2-infected hamsters. Comparison of body weight change (**a**) in LD (10^2.5^ PFU, *n* = 30), HD (10^5^ PFU, *n* = 15), and CP-LD (immunocompromised; 10^2.5^ PFU, *n* = 30) SARS-CoV-2-infected hamsters over 2–16 dpi. Median weight change (g) over 2–16 dpi, compared to weight at the time of infection. Comparison of viral load (**b**–**f**) in SARS-CoV-2-infected hamsters (LD vs. HD, and LD vs. CP-LD) at 2–16 dpi, expressed as PFU/g of tissues. The expression of SARS-CoV-2 N gene copies/g of lungs over 2–16 dpi in LD, HD, and CP-LD-infected hamsters (**g**). Kinetics of plaque reduction neutralization titer in hamster sera collected at 2–16 dpi (**h**). Data represent mean ±SD. and each dot represents data from an individual hamster. LD and CP-LD; *n* = 4–6, HD, *n* = 3 per time point; only animals that showed a positive response were included in the plots. Statistical analysis was performed by one-way ANOVA with Tukey’s multiple group comparisons. ** *p* < 0.01; *** *p* < 0.005. Significant *p*-values in (**b**–**g**) are indicated above the plots.

Following intranasal infection, hamsters from all groups survived until the experimental euthanasia. Compared to the uninfected, healthy animals, both the LD and HD-infected immunocompetent hamsters showed a reduction in body weight from the day of infection until six days post-infection (dpi). The animals gradually gained weight from 8 to 16 dpi, suggesting that they experienced inappetence or anorexia during the early stages of the disease, as determined by food/water intake. A maximum mean weight loss of 2.63% and 8.4%, respectively, was observed in LD and HD infection groups at 6 dpi (Figure 1a). To model immunosuppressed host conditions, a group of hamsters was treated with CP (70 mg/kg) before and during LD intranasal SARS-CoV-2 infection (see Methods). Previous reports indicate that hamsters treated with CP at this dose had about a 15-fold reduction in the total white blood cells, a surrogate of immunosuppression [17]. In contrast to the immunocompetent animals, the immunocompromised (CP-LD) hamsters showed slow weight loss up to 8 dpi from the day of infection. These animals also showed a slow-weight gain between 8 and 16 dpi (Figure 1a). The maximum mean weight loss observed was 7.67% at 8 dpi. However, the mean body weight on day 16 was only 2.8% higher than the mean weight at infection (Figure 1a).

### 3.2. Viral Burden in Immunocompetent Hamsters Infected with a Low or High Dose Inoculum

In both LD and HD-infected hamsters, the highest viral burden was detected in the lungs, turbinate, and larynx/trachea at 2 dpi (Figure 1b). The viral load in these organs did not significantly change between 2 and 4 dpi. At this time point, infectious viral particles were also detected in the heart, adrenal gland, epididymal fat pad, brain (including olfactory bulb), and eyes of both LD and HD-infected animals (Figure 1b,c). However, at both 2 and 4 dpi, the viral load in these extrapulmonary organs was about 3–4 logs, significantly lower than in the lungs (Figure 1b,c).

In the LD-infected hamsters, viruses were detected in the colon, kidney, and adrenal glands of one out of six animals at 2 and 4 dpi in the liver and spleen. In contrast, at 4 dpi, none of the hamsters had viruses in the colon and kidney, and three had viruses in the adrenal glands (Figure 1b,c, Table 1). Four out of six hamsters at 2 dpi and all six at 4 dpi had infectious viral particles in the heart. However, the viral load in the extrapulmonary organs was not significantly different between 2 and 4 dpi (Figure 1b,c). Four out of six hamsters at 7 dpi showed viral load in the nasal turbinate, while one animal each showed viruses in the lungs and nasal lavage (Figure 1d, Table 1). The viral load in these tissues was significantly lower at 7 dpi than at 2 and 4 dpi (Figure 1b–d).

Following HD infection, the peak viral load was detected at 2 and 4 dpi. Two out of three hamsters had viruses in the heart, spleen, adrenal, epididymal fat, and brain at 4 dpi. At this time, one out of three hamsters showed viral load in the liver, kidney, and colon (Figure 1c). At 7 dpi, the infectious virions were detectable in the lungs and turbinates of one out of three hamsters (Figure 1d). The viral loads in the lung, nasal lavage, nasal turbinates, larynx/trachea, heart, epididymal fat, and brain were not significantly different between LD and HD at 2 and 4 dpi (Figure 1b,c). To summarize, the viral burden in pulmonary and extrapulmonary organs was not significantly different between LD and HD SARS-CoV-2-infected immunocompetent hamsters at 2, 4, 7, 12, and 16 dpi.

### 3.3. Disparate Tissue Viral Burden between Immunocompetent and Immunocompromised Hamsters Infected with SARS-CoV-2

Next, we examined the effect of immune suppression on SARS-CoV-2 replication and time to viral clearance in tissues. In contrast to LD-infected immunocompetent hamsters, the immunocompromised animals had infectious SARS-CoV-2 virions in their respiratory tract from 2 dpi until 16 dpi (Figure 1b–f). In these animals, the viral load in the nasal lavage gradually decreased from 2 to 7 dpi and stabilized at similar levels up to 16 dpi (Figure 1b–f). The viral load peaked in the lungs at 4 dpi in the immunocompromised hamsters (CP-LD) (Figure 1c), then gradually declined between 7 dpi to 12 dpi (Figure 1d,e). A similar viral load was observed between 12 and 16 dpi (Figure 1e,f). In contrast, the peak viral load was noted at 2 dpi in the nasal turbinates and larynx/trachea, which gradually declined at 7 dpi (Figure 1b,d). The viral load was not significantly different in these organs at 7, 12, and 16 dpi. The infectious virus in the heart was detectable at 2, 4, and 7 dpi that declined at 12 dpi (one out of six animals had the virus) before rebounding at 16 dpi (five out of six had a detectable virus) (Figure 1b–f). All the immunocompromised and infected hamsters had infectious viruses in the adrenal gland, brain, eye, colon, bone marrow, liver, and spleen; five out of six animals in kidneys and four out of six in epididymal fat at 2 dpi (Figure 1b). The mean viral load in the adrenal gland, bone marrow, liver, and colon declined gradually from 2 until no live virus was detected in 7 dpi (Figure 1b–d). At 12 dpi, the virus was detected only in the heart (one out of six), eyes (three out of six), and respiratory system (Figure 1e). Interestingly, infectious viruses were detected in the heart, adrenal gland, epididymal fat, brain, eye, and bone marrow of some immunocompromised and infected hamsters at 16 dpi (Figure 1f).

No significant difference in the viral load was observed in the nasal lavage, turbinates, larynx/trachea, heart, adrenal gland, brain, eyes, and bone marrow between immunocompetent (LD) and immunosuppressed (CP-LD) animals at 2 and 4 dpi. However, a significantly higher viral load was noted in the lungs of infected immunocompromised hamsters at 2 and 4 dpi and in the nasal lavage of immunocompetent hamsters at 2 dpi. Similarly, the live virus was detected in the liver and spleen of only immunocompromised hamsters at 2 dpi and in the kidneys at 4 dpi. Replicating viral load was observed in the heart, adrenal, epididymal fat pad, brain, eyes, and bone marrow in these animals at 7 and 16 dpi (Figure 1b–f).

The total viral burden in the lung was also determined by measuring the SARS-CoV-2 N gene (+and—strand RNA) transcripts, which were detected in the lungs of immunocompromised and immunocompetent infected hamsters (both LD and HD) from day 2 to 16 dpi. A peak in viral RNA load was observed in LD and HD groups at 2 and 4 dpi, although the viral RNA load was not significantly different between these two groups at 2, 4, 7, 12, and 16 dpi (Figure 1g). Interestingly, although more infectious viruses were detected in the infected immunocompromised hamster lungs from day 2 to 16 dpi, the viral RNA load was not significantly different from the immunocompetent (LD) group (Figure 1g).

### 3.4. Heterogeneity in Neutralizing Antibody Titer in SARS-CoV-2-Infected Immunocompetent and Immunocompromised Hamsters

The plaque reduction neutralization test (PRNT) revealed that both LD and HD SARS-CoV-2-infected hamsters elicited detectable virus-neutralizing antibodies at 7 dpi. At 7 and 16 dpi, LD-infected hamsters showed 90% PRNT neutralization (PRNT_90_) at 1:160 to 1:640 dilutions, respectively. The HD-infected hamsters showed PRNT_90_ at 1:360 to 1:640 serum dilutions at these time points (Figure 1h). Notably, the sera from immunocompromised hamsters showed PRNT_90_ at <1:16 dilution except for one hamster serum at 7 dpi, which showed a PRNT_90_ at 1:64 dilutions (Figure 1h). These observations suggest that SARS-CoV-2 neutralizing antibody production was reduced in immunocompromised hamsters. Further, the presence of viable SARS-CoV-2 until 16 dpi in the infected immunocompromised animals, as well as a significant reduction or absence of infectious viruses starting at 7 dpi in LD and HD-infected immunocompetent hamsters, indicate that the extent and rapidity of SARS-CoV-2 clearance are associated with the levels of production of virus-neutralizing antibodies at the systemic level.

### 3.5. Cytokine Storm Marker Expression in SARS-CoV-2-Infected Immunocompetent and Immunocompromised Hamsters

**Figure 2:**
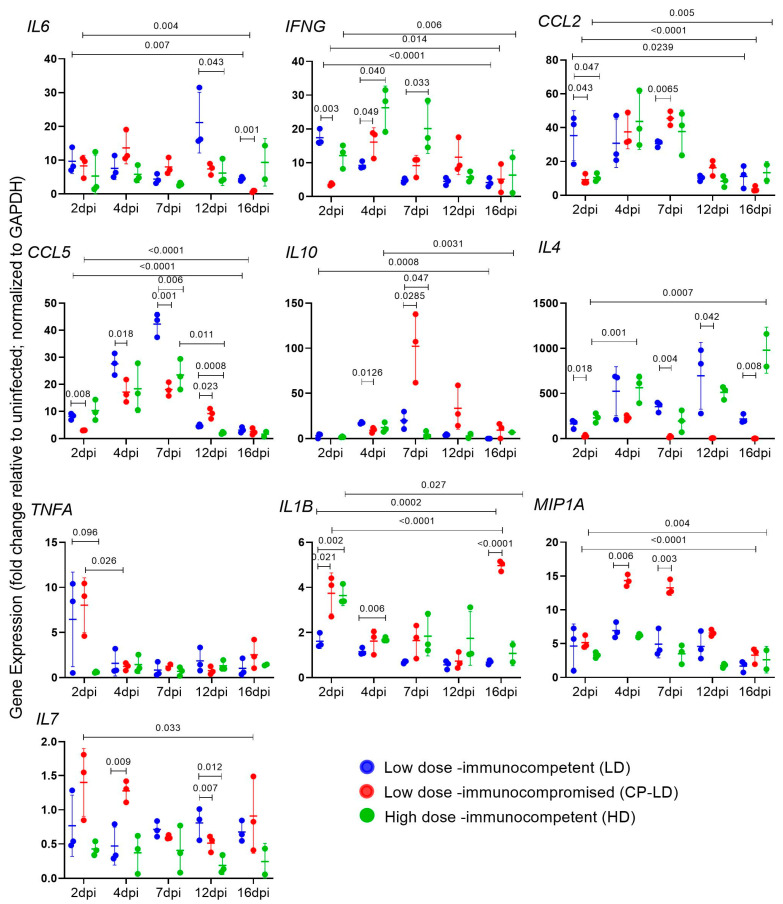
Expression of pro and anti-inflammatory cytokines/chemokines in SARS-CoV-2-infected hamster lungs. Comparative expression levels of IL6, IFNγ, CCL2, CCL5, IL10, IL4, TNFα, IL1β, MIP1α, and IL7 gene transcripts in immunocompetent LD (blue) and HD (green), or immunocompromised LD (CP-LD) SARS-CoV-2-infected hamster lungs collected at 2, 4, 7, 12, and 16 dpi. (*n* = 3 for each time point and condition repeated 3 times; each dot represents the average of 3 technical replicates). The gene expression levels of target genes were normalized to GAPDH transcript levels and represent the fold change in expression compared to uninfected hamsters. Statistical analysis was performed using an unpaired Student’s *t*-test with Welch correction. Data represent mean ± SD.

Both LD and HD-infected hamsters showed more than 10-fold induction of *IL6* (Figure 2), *IFNG* (Figure 2), *CCL2* (Figure 2), *CCL5*(Figure 2), *IL10* (Figure 2), *IL4* (Figure 2), and a moderate increase in *TNFα* (Figure 2), *IL1B* (Figure 2), and *MIP1A* (Figure 2) transcripts over 2–16 dpi, compared to the uninfected hamsters. The expression of *IL7* transcripts was downregulated at all the tested time points (Figure 2). The SARS-CoV-2-infected immunocompromised (CP-LD) hamsters showed more than a 10-fold increase in the expression of *IL6* (Figure 2), *IFNG* (Figure 2), *CCL2* (Figure 2), *CCL5* (Figure 2), *IL10* (Figure 2), *IL4* (Figure 2), *MIP1A* (Figure 2), and a slight increase in the levels of *TNFA* (Figure 2), and *IL1B* (Figure 2). The level of TNFα expression was not significantly different between LD and HD-infected, as well as immunocompromised and immunocompetent hamster groups at day 2 to 16 pi (Figure 2). The level of *CCL5* expression was higher in LD than in the HD and CP-LD-infected hamsters at 7 dpi (Figure 2). While the expression of *IL4* (Figure 2) and *MIP1A* (Figure 2) were similar in both LD and HD, the infected immunocompromised hamsters showed 10–60-fold lower *IL4* (Figure 2) and 2-fold higher *MIP1A* (Figure 2) expression than LD. The expression of *IL1B* was higher in the lungs of HD than in LD-infected hamsters at 2, 4, and 7 dpi (Figure 2). In comparison, the infected immunocompromised hamsters showed elevated levels of *IL1B* at 2 and 16 dpi (Figure 2). The level of *IL6* expression was significantly higher 12 dpi in LD than in HD-infected hamsters (Figure 2). The infected immunocompromised (CP-LD) hamsters also had comparable levels of *IL6* expression to LD except at 12 and 16 dpi (Figure 2). CCL2 expression was higher in LD than HD at 2 dpi (Figure 2), while infected immunocompromised hamsters showed elevated *CCL2* transcript levels compared to the LD group at 4 and 7 dpi (Figure 2). Although *IL10* expression was similar between LD and HD-infected hamsters (Figure 2); it was higher in infected immunocompromised hamsters at 7 dpi (Figure 2). IFNγ was high in HD than LD at 4 and 7 dpi (Figure 2); however, CP-LD showed slow induction and increased expression at 4 to 12 dpi compared to LD (Figure 2).

### 3.6. Severity of Pulmonary/Bronchiolar Pathology and Thrombosis in SARS-CoV-2-Infected Immunocompetent and Immunocompromised Hamsters

**Figure 3:**
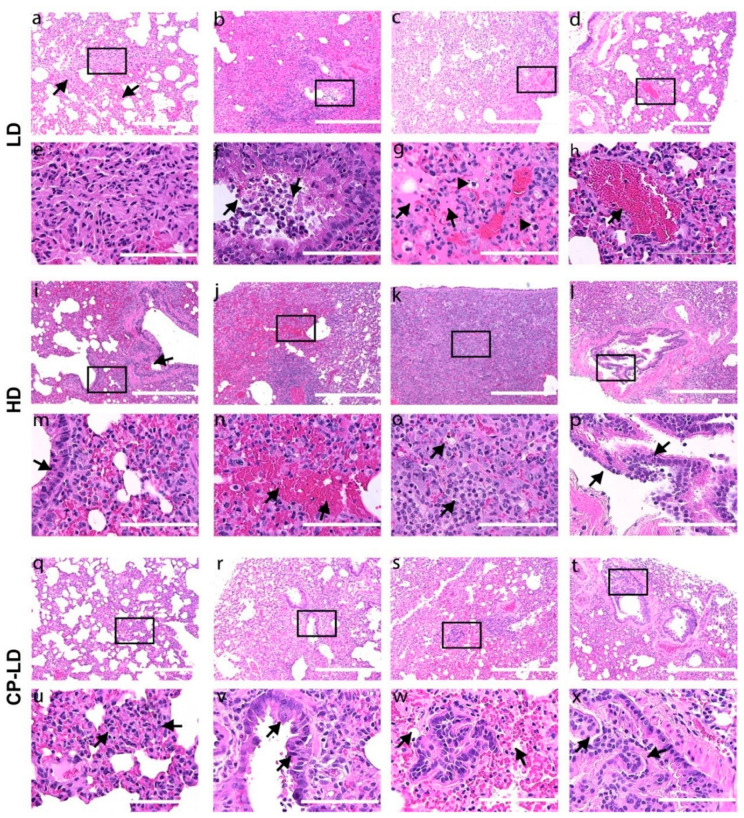
Interstitial pneumonia, bronchioalveolar, and vascular pathology in SARS-CoV-2-infected hamsters. Histopathological analysis of LD, HD, and CP-LD-infected hamster lungs revealed infiltration of mononuclear cells in the interstitium (**a**–**h**; arrows in **a**), in the bronchiolar lumen (**b**,**f**; arrows in **f**), alveolar and interstitial edema (**c**,**g**; arrows in **g**), foamy macrophages (arrows heads in **g**) and early thrombus (**c**,**d**,**g**,**h**; arrow in **h**), interstitial/alveolar capillary congestion (**b**) of LD-infected hamsters. Bronchiolar hyperplasia (**i**,**l**,**m**,**p**; arrow in **i**), alveolar epithelial hyperplasia (**i**,**m**; arrow in **m**), interstitial hemorrhages (**j**,**n**; arrows in **n**), and severe infiltration of inflammatory cells in the alveoli and interstitium caused the obliteration of alveoli in HD-infected hamsters. Foamy macrophages (**j**,**k**,**n**,**o**; arrows in **o**), bronchiolar epithelial hyperplasia and detachment, bronchiolitis, and bronchiolar smooth muscle hyperplasia (**l**,**p**; arrows in **p**) were also noted in HD-infected hamsters. Moderate infiltration of mononuclear cells and congestion of the interstitium (**q**–**x**; arrows in **u**), multiple alveolar epithelial hyperplasia (arrows in **v**), and obliteration of alveoli (**r**,**s**,**v**,**w**), severe bronchiolar epithelial hyperplasia, and obliteration of lumen (**t**,**x**) were observed in the immunosuppressed infected (CP-LD) hamsters. Images (**a**–**d**,**i**–**l**,**q**–**t**) are 100× magnifications, and (**e**,**f**,**m**–**p**,**u**–**x**) are 400× magnifications (marked in 100× images). Scale bar represents 100 µ (**e**,**f**,**m**–**p**,**u**–**x**) or 400 µ (**a**–**d**,**i**–**l**,**q**–**t**). Note: panels (**a**,**b**,**e**,**f**,**i**,**j**,**m**,**n**,**q**,**t**,**u**,**x**) are 4 dpi, (**c**,**g**,**k**,**o**,**r**,**v**) are 7 dpi, and (**d**,**h**,**j**,**p**,**s**,**w**) are 16 dpi.

In both LD (Figure 3a–h) and HD (Figure 3i–p) groups, the lungs showed multifocal to diffuse infiltration of mononuclear cells in the interstitial spaces with the maximum infiltration 4 (Figure 3a,b,e,f,i,j,m,n) and 7 dpi (Figure 3c,d,g,h,k,l,o,p). The inflammatory cellular infiltrations obliterated the alveoli and resulted in alveolar collapse (Figure 3b,c,j,k). A lower degree of inflammatory cell infiltration was noted in the immunocompromised animals 4 and 7 dpi (Figure 3r,t) compared to immunocompetent hamsters infected with LD SARS-CoV-2 (Figure 3b,c,s,w). The pulmonary parenchyma of immunocompetent hamsters infected with LD SARS-CoV-2 showed moderate levels of mononuclear cells infiltration in the interstitium (Figure 3a,b,e and Table 2), congestion of capillaries in the alveolar wall (Figure 3a,b), mild bronchiolar epithelial hyperplasia (Figure 2), bronchiolitis with lymphocytes infiltration, and necrotic bronchiolar epithelial cells in the bronchiolar lumen (Figure 3b,f) at 4 dpi. In these animals, the presence of foamy macrophages and interstitial edema was prominent at 7 dpi (Figure 3c,g).

The immunocompetent hamsters infected with HD SARS-CoV-2 had severe multifocal to diffused mononuclear cell infiltration in the interstitium at 4 dpi (Figure 3i,j,m,n). At this time, extensive bronchiolar epithelial hyperplasia with the detachment of hyperplastic epithelial cells and lymphocyte infiltration in the lumen (Figure 3i) and alveolar epithelial hyperplasia wherein proliferation of type II alveolar cells was noted (Figure 3i,m). In addition, multifocal edema of the interstitium and alveoli, congestion of capillaries in the alveolar walls (Figure 3i,m), focal hemorrhages (Figure 3j,n), and foamy macrophages (Figure 3n) (one in three hamsters) were observed (Figure 2 and Table 2). Trichrome staining of the lung sections showed multifocal thickening of alveolar walls. At 7 dpi, severe pneumonia appeared as a pseudolymphoid tissue (Figure 3k,o), and the infiltration of foamy macrophages (Figure 3o) in the pulmonary interstitial connective tissue was observed. At 7 and 16 dpi, lung sections showed “forming thrombi or early thrombi” in the arterioles (Figure 3g,h). However, a lesser degree of pulmonary interstitial pneumonia was noted at 16 dpi (Figure 3d,h,l,p) than at 7 dpi (Figure 3c,g,k,o and Table 2). Overall, the degree of interstitial pneumonia and bronchiolar-alveolar hyperplasia was severe in HD SARS-CoV-2-infected hamsters compared to LD-infected hamsters.

The immunocompromised SARS-CoV-2-infected hamsters (CP-LD) showed a lower number of inflammatory cells (Figure 3q–x) and edema in the interstitium (Figure 2) than the immunocompetent/LD-infected animals. In the CP-LD group, mild infiltration of foamy macrophages was observed at 7 dpi (Figure 3v). However, CP-LD hamsters revealed severe hyperplasia of bronchiolar-alveolar epithelial cells (Figure 3r,v,s,w) with the occlusion of alveolar (Figure 3s,w) and bronchiolar lumen (Figure 3t,x) were observed throughout the lung parenchyma (Table 2).

The trachea of LD and HD-infected immunocompetent hamsters revealed multifocal denudation of mucosal epithelial cells into the lumen, metaplasia of tracheal epithelium, hyperplastic goblet cells, infiltration of lymphocytes and neutrophils in the lumen and submucosa, and mucous exudate in the tracheal lumen at 4 and 7 dpi (Figure S1). On day 16, elevated mononuclear and neutrophil infiltration was noted in the LD group (Figure S1a,d). However, the degree of neutrophil infiltration in the submucosa was higher, with some hamsters showing a complete detachment of the mucosa-submucosal layer from the cartilage in the HD group (Figure S1b,e). In contrast, severe hyperplasia and necrosis of mucosal epithelia, severe infiltration of neutrophils, and mild lymphocytes infiltration in the submucosa were observed in the CP-LD hamsters as early as 4 dpi (Figure S1c and Figure 1f). In these animals, multifocal necrosis and detachment of mucosal and submucosal layers were observed at 7 and 16 dpi (Figure S1c and Figure 1f). Immunocompromised (CP-LD) hamsters had a lesser degree of inflammatory cell infiltration and the delayed resolution of inflammation in the lungs than LD-infected hamsters. Meanwhile, CP-LD hamsters showed more severe bronchiolar-alveolar hyperplasia than LD-infected hamsters. It indicated that the disease is prolonged in CP-LD-infected hamsters, as observed in body weight change.

Consistent with the histopathologic findings, qPCR analysis of B and T cell lineage markers in LD and CP-LD-infected hamster lungs revealed upregulated expression of markers of the B cell (CD22) and plasma cell markers (CD138) and the Treg marker (CD25) was significantly downregulated in LD, compared to CP-LD-infected hamster lungs (Figure S2). However, the levels of T cell markers, CD3, CD4, and CD94, expression in the lungs were not significantly different between LD and CP-LD-infected animals at 7 dpi (Figure S2).

### 3.7. Hypertension-like Vascular Smooth Muscle Hyperplasia/Hypertrophy in the Lungs and Kidneys of SARS-CoV-2-Infected Hamsters

**Figure 4:**
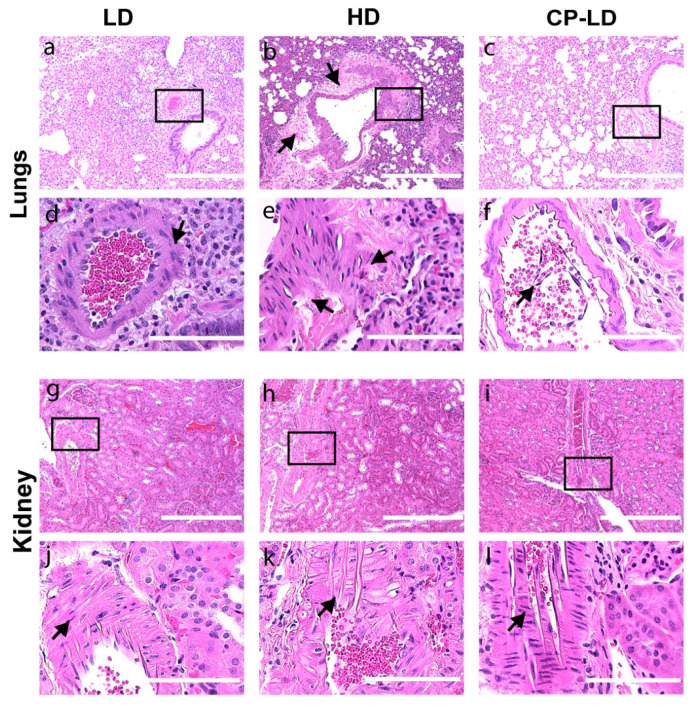
Arteriolar pathology in lungs and kidneys of SARS-CoV-2-infected hamsters. Histopathological analysis of pulmonary parenchyma revealed mild arteriolar smooth muscle hyperplasia (**a**), severe perivasculitis and smooth muscle hyperplasia (**b**; arrows), detachment of endothelial cells into the arteriolar lumen, and mild perivasculitis (**c**) in LD, HD, and CP-LD-infected hamsters, respectively. The images (**d**–**f**) are a magnification of the boxed area in (**a**–**c**), respectively. Moderate (LD and CP-LD) (**g**,**i**) to severe (HD) (**h**) vascular smooth muscle hyperplasia in the renal arterioles in SARS-CoV-2-infected hamsters. The images in (**j**,**k**,**l**) are a magnification of the boxed areas in (**g**–**i**), respectively. The images (**a**–**c**,**g**–**i**) are 100×, and (**d**–**f**,**j**–**l**) are 400× magnifications (marked in 100× images). Scale bar represents 100 µm (**d**–**f**,**j**–**l**) or 400 µm (**a**–**c**,**g**–**i**).

We observed the thickening of the vascular smooth muscle layer/tunica media in the histology sections of the lungs (Figure 4a–f) and kidneys (Figure 4g–l) of immunocompetent animals infected with LD (Figure 4a,d,g,j) and HD (Figure 4b,e,h,k) at 7 dpi. Two of the immunocompetent/HD-infected hamsters showed hypertrophy of the muscular layer in the arteriole and mononuclear cells infiltration in the adventitial layer of the arteriole (perivasculitis) and proliferation of tunica externa (Figure 4b,e). In contrast, in the immunocompromised LD-infected hamsters, severe vascular lesions, denudation of endothelial cells into the lumen, mononuclear cells infiltration in the adventitia of small blood vessels, and rupture of the arteriolar wall, including muscular layer and leakage of vascular contents, was noted at 7 dpi (Figure 4c,f).

The LD, HD, and CP-LD-infected hamsters had severe adrenal cortical and medullary degeneration, necrosis, and liquefaction (Figure S2a–l) with cortical hypertrophy in some hamsters (Figure S2e,f). A moderate-to-severe arteriolar smooth muscle hypertrophy and hyperplasia were also observed in the renal arterioles (Figure 4g–l). Thus, in addition to the hypertrophy/hyperplasia of vascular smooth muscles and adrenal cortical pathology, which are reported to be associated with hypertension, the thickening of vascular smooth muscles can also be an indication of hypertension induced by SARS-CoV-2.

### 3.8. Multi-Organ Pathology Induced by SARS-CoV-2 Infection in Immunocompetent and Immunocompromised Hamsters

Consistent with the clinical studies [21], the spleens of immunocompetent LD and HD SARS-CoV-2-infected hamsters showed a reduction in size and number of white pulp compared to uninfected hamsters at 4 dpi (Figure 4g,h and Figure S3). In the infected animals, white pulp lymphocytes were replaced by trabecular connective tissues. In contrast, the immunocompromised LD SARS-CoV-2-infected hamsters (CP-LD) showed a high degree of white pulp atrophy at all the time points tested (4, 7, and 16 days post-infection) (Figure 4i,l).

The kidneys of immunocompetent hamsters infected with LD or HD showed mild lymphocyte infiltration in the interstitial tissues and multifocal tubular degeneration, acute tubular necrosis with the detachment of tubular cells into lumen, tubular epithelial cells degeneration with pyknotic nuclei or karyorrhexis, or chromatolysis at 4, 7, and 16 dpi (Figure S4a–h). In these animals, focal regions of tubulointerstitial edema were also observed. The degree of acute tubular necrosis was higher in hamsters infected with HD than in LD (Figure S4d,f). At 7 (two out of three hamsters) and 16 dpi (three hamsters), a moderate renal arteriolar media (smooth muscle) hyperplasia/hypertrophy was observed in the LD-infected hamsters (Figure S4g,j). These pathological manifestations appeared early (4 dpi) in the HD-infected animals (Figure S4h,k).

The kidneys of CP-LD hamsters showed lymphocyte infiltration in the interstitial tissues, focal/multifocal acute tubular necrosis with the detachment of tubular epithelial cells from the basement membrane (Figure S4i,j), forming concentric layers of eosinophilic masses with necrotic nuclei in the tubular lumen (Figure S4k,l), at 4, 7, and 16 dpi. However, the degree of tubular necrosis in these animals was less compared to the kidneys of immunocompetent LD-infected hamsters. Further, the arteriolar media hyperplasia/hypertrophy was less prominent in these animals (Figure 4i,l). The CP-LD hamsters also showed basophilic ground-glass bodies in the tubular epithelial cells and glomerular degeneration at 4, 7, and 16 dpi (Figure S4k,l).

**Figure 5:**
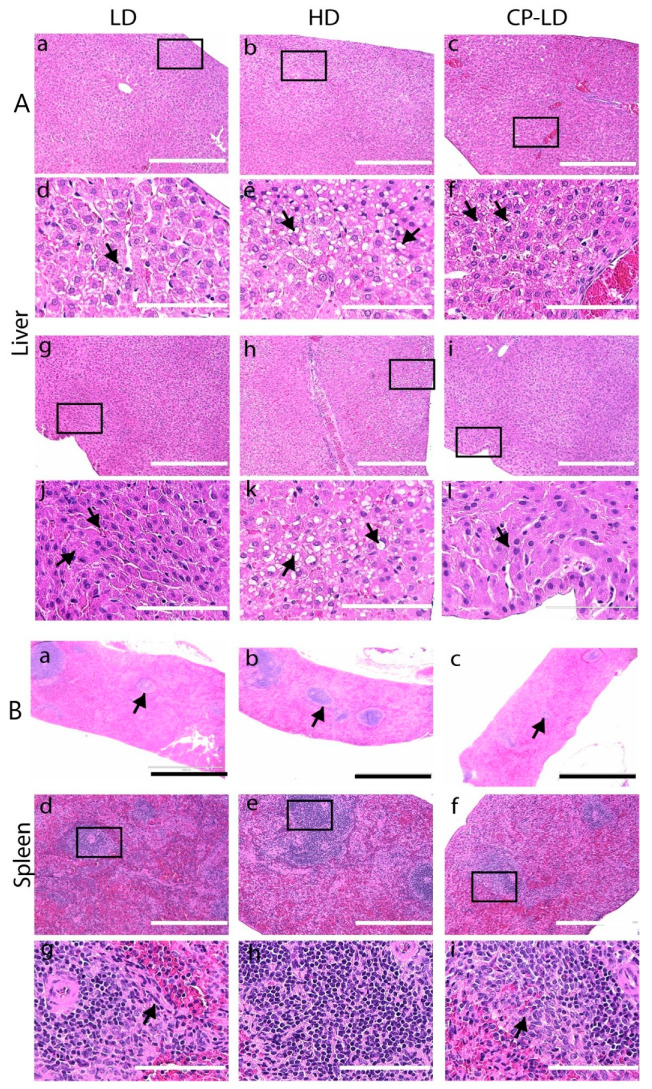
Histopathology of liver (**A**) and spleen (**B**) in SARS-CoV-2-infected hamsters. (**A**): Histopathological analysis of LD, HD, and CP-LD-infected hamster liver (4, 7, and 16 dpi, *n* = 3 per time point) showed the infiltration of lymphocytes in sinusoids at 4 dpi (**a**,**d**), and pyknosis (arrows in **d**), karyorrhexis, and karyolysis in necrotic hepatocytes (**g**, arrows in **j**) at 7 dpi. Necrotic hepatocytes with pyknotic nuclei and mild to moderate steatosis (**b**, arrows in **e**) and severe congestion in the portal vein (**h**) were noted at 4 dpi, while severe congestion in the portal vein, sinusoids, and mild steatosis (**c**, arrows in **k**) were observed at 7 dpi in HD-infected hamsters. Severe degeneration and necrosis of hepatocytes near the parietal surface were observed at 4 dpi in CP-LD animals (**i,** arrows in **l**). Images (**a**–**c**,**g**–**i**) are 100×, and (**d**–**f**,**j**–**l**) are 400× magnifications. Scale bar represents 100 µm (**d**–**f,j**–**l**), or 400 µm (**a**–**c**,**g**–**i**). (**B**): Histopathological analysis of the spleen revealed a reduction in the number and area of white pulp lesions and cellular composition with increased trabecular connective tissues at 4 dpi in LD (**a**,**d**,**g**), HD (**b**,**e**,**h**), and CP-LD (**c**,**f**,**i**)-infected hamsters. Images (**a**–**c**) are 40×, (**d**–**f**) are 100×, and (**g**–**i**) are 400× magnifications. Scale bar represents 100 (**g**–**i**), 400 (**d**–**f**), or 1000 µm (**a**–**c**).

Since steatosis (vacuolation) of hepatocytes is commonly observed among COVID-19 patients [22], we investigated these pathological features in the SARS-CoV-2-infected hamsters. Liver histology revealed the diffuse infiltration of a few lymphocytes and neutrophils in LD, HD, and CP-LD-infected hamsters (Figure 5A(a–l)) and degeneration of hepatocytes with pyknosis at 4 and 7 dpi (Figure 5). In addition, mild steatosis and portal vein congestion was observed at 16 dpi (not shown). The LD-infected hamsters showed mild steatosis (Figure 5A(a,d,g,j)); in contrast, immunocompetent HD SARS-CoV-2-infected hamsters showed moderate-to-severe multifocal to diffuse steatosis (Figure 5A(b,e)) and portal vein congestion at 4, 7, and 16 dpi (Figure 5A(h,k)).

In contrast, in the CP-LD group, one of the three hamsters at 4 and 7 dpi and all hamsters at 16 dpi had severe liver degeneration, marked with eosinophilic granular cytoplasm and pyknosis (Figure 5A(i,l)). In these animals, mild-to-moderate steatosis, portal vein congestion, and edema in the sinusoids surrounding the portal vein were observed (Figure 5A(c,f)).

### 3.9. SARS-CoV-2 Host Cell Entry Receptor Expression in Immunocompetent and Immunocompromised Hamster Lungs

**Figure 6:**
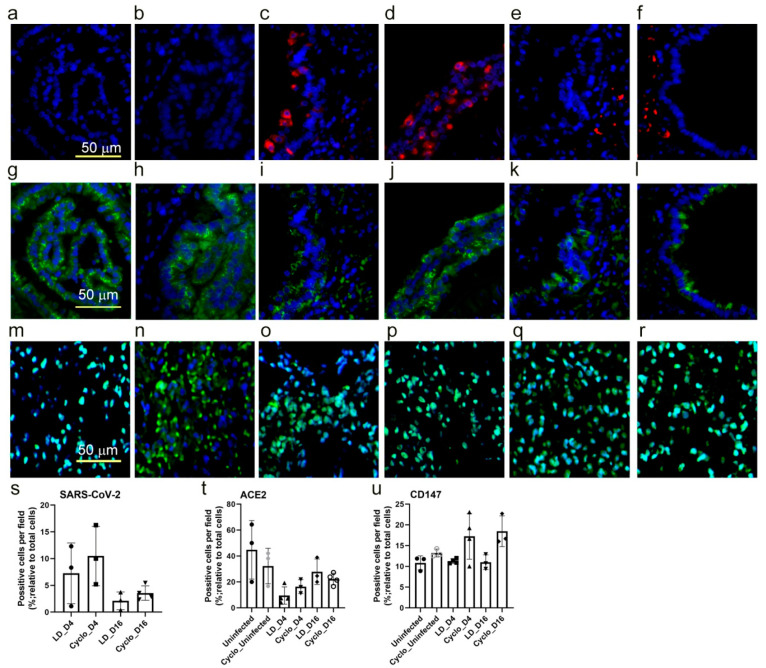
Spatial expression of SARS-CoV-2 host cell entry receptors in immunocompetent and immunocompromised hamsters. Lung sections were stained for SARS-CoV-2 (**a**–**f**), ACE-2 receptor (**g**–**l**), or CD147 (**m**–**r**). Images (**a**,**g**,**m**) are uninfected immunocompetent hamster lung sections, and (**b**,**h**,**n**) are uninfected immunocompromised hamster lung sections. Images (**c**,**i**,**o**) are SARS-CoV-2-infected immunocompetent hamster lung sections at 4 dpi; and (**d**,**j**,**p**) are SARS-CoV-2-infected immunocompromised hamster lung sections at 4 dpi. Images (**e**,**k**,**q**) are SARS-CoV-2-infected immunocompetent hamster lung sections at 16 dpi; and (**f**,**l**,**r**) are SARS-CoV-2-infected immunocompromised hamster lung sections at 16 dpi. The red color in (**c**–**f**) indicates the presence of SARS-CoV-2, and the green color indicates ACE-2 receptor expression in (**g**–**l**) or CD147 expression in (**m**–**r**). Image (**s**) shows lung cells positive for SARS-CoV-2 in immunocompetent (LD) and immunocompromised (Cyclo) hamsters at 4 (D4) and 16 dpi (D16); image (**t**) shows lung cells positive for ACE-2 in uninfected and SARS-CoV-2-infected immunocompetent (LD) and immunocompromised (Cyclophosphamide-treated) hamsters at 4 (D4), and 16 dpi (D16) and (**u**) shows lung cells positive for CD147 in uninfected and SARS-CoV-2-infected immunocompetent (LD) and immunocompromised (Cyclophosphamide-treated) hamsters at 4 (D4) and 16 dpi (D16). *n* = 3 per group.

We observed a slightly elevated level of SARS-CoV-2 N protein in the immunocompromised compared to immunocompetent infected hamsters at 4 and 16 dpi (Figure 6c–f), though the difference in the number of cells positive for the SARS-CoV-2 N gene was not statistically significant (Figure 6s). At both 4 and 16 dpi, ACE2 receptor expression was insignificantly lower in the infected immunocompetent and immunocompromised hamsters compared to the respective uninfected control groups (Figure 6g–l,t). In contrast, CD147 expression was higher in the infected immunocompromised hamsters at both 4 and 16 dpi compared to the infected and uninfected immunocompetent animals. However, the difference was not statistically significant between these groups (Figure 6m–r,u). Together, the data suggest that key SARS-CoV-2 host cell entry receptors (ACE-2 and CD147) show distinct expression patterns between immunocompetent and immunocompromised hamster lungs, although the difference in expression pattern did not correlate with the disease pathology in respective infection groups.

### 3.10. SARS-CoV-2 Infection Elicits Distinct Transcriptome Profiles in the Lungs

**Figure 7:**
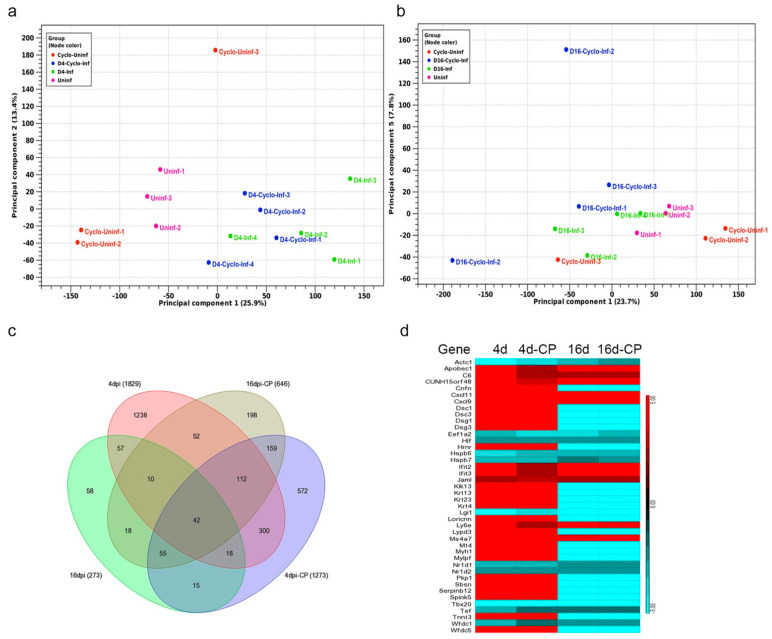
Genome-wide lung transcriptome analysis of immunocompetent and immunocompromised hamsters infected with SARS-CoV-2. (**a**) PCA plot of immunocompetent and immunocompromised hamsters without infection at 4 dpi. (**b**) PCA plot of immunocompetent and immunocompromised hamsters without infection at 16 days post SARS-CoV-2 infection. (**c**) Venn diagram showing the number of significantly differentially expressed genes (SDEG) in the lungs of immunocompetent and immunocompromised hamsters at 4 or 16 dpi. Data from the infected animals were normalized to corresponding uninfected animal data. (**d**) Heat-map of SDEGs commonly perturbed in all four groups. Scale bar shows up (red) and downregulation (cyan). *n* = 3 per group.

The principal component analysis (PCA) showed the segregation of uninfected groups from those at different stages of infection with or without immunosuppression (Figure 7a,b).

Analysis of differentially expressed genes showed a greater number of significantly differentially expressed genes (SDEG) at 4 dpi than 16 dpi in both immunocompetent and immunocompromised animals upon SARS-CoV-2 infection (Figure 7c). However, the number of SDEGs in the immunosuppressed animals was less than in immunocompetent animals at 4 dpi (1273 versus 1829). In contrast, the former group had more SDEGs at 16 dpi (646 versus 273). Thus, immunocompetent and immunocompromised hosts showed distinct gene expression profiles at different stages of infection (i.e., 4 dpi/acute and 16 dpi/chronic stages). Among the 4 groups (i.e., 4 and 16 dpi in immunocompromised and immunocompetent groups), 42 common SDEGs were identified. Of these, 31 were upregulated in the immunocompetent group, compared to 10 SDEGs in the immunocompromised group at 4 and 16 dpi, respectively (Figure 7d). Furthermore, 50% of the 42 SDEGs were expressed in opposite directions between the immunocompetent and immunocompromised groups at 4 and 16 dpi.

### 3.11. SARS-CoV-2 Infection Elicits Distinct Acute and Chronic Transcriptome Profiles in the Lungs of the Immunocompetent Host

**Figure 8:**
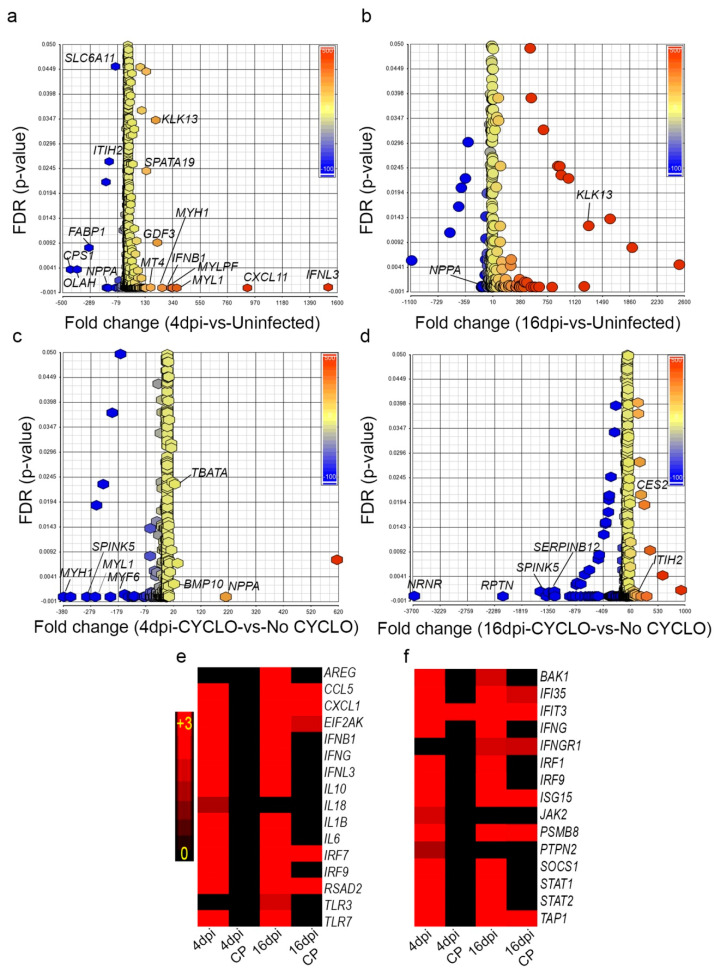
Distribution of differentially expressed genes in immunocompetent and immunocompromised hamsters infected with SARS-CoV-2. (**a**) Scatter plot of SDEG in immunocompetent hamster lungs at 4 dpi. (**b**) Scatter plot of SDEG in immunocompetent hamster lungs at 16 dpi. (**c**) Scatter plot of SDEG in immunocompromised hamster lungs at 4 dpi. (**d**) Scatter plot of SDEG in immunocompromised hamster lungs at 16 dpi. (**e**) Heat-map of SDEG involved in the hypercytokinemia/chemokinemia network. (**f**) Heat-map of SDEG involved in the canonical IFN signaling pathway. Scale bar shows upregulation (red) and no expression (black). FDR—false discovery rate.

Canonical pathways upregulated at 4 dpi strongly suggests the induction of a classical innate and proinflammatory host response. This includes natural killer (NK) cell signaling, hypercytokinemia/chemokinemia, pattern recognition receptor (PRR) signaling, dendritic cell (DC) maturation, communication between DC and NK cells, and the Th1 pathway (Figure S5). Interferon (IFN) lambda-3 and IFN beta-1 were among the top upregulated genes in this group (Figure 8a–d). In contrast, the top canonical pathways perturbed at 16 dpi involved tissue thrombosis, such as intrinsic prothrombin activation, MSP-RON signaling in macrophages and coagulation system pathways (Figure S6). Other pathways involved in host cell activation, including LXR/RXR activation, production of reactive oxygen and nitrogen intermediates in macrophages, and acute phase response signaling, were significantly dampened at this time when the animals recovered from viral infection (i.e., no live virus) and gained body weight. Thus, the genome-wide lung transcriptome profile is consistent with the corresponding pathophysiological manifestations observed during acute (4 dpi) and chronic (16 dpi) stages of infection. Notably, the expression of the SDEGs involved in hypercytokinemia/chemokinemia (e.g., *CCL5, IFNB1*, *IFNG, IFNL3, IL1B, IL1, IL18, IRF7,* and *IRF9*) and interferon signaling pathways (e.g., *CXCL9*, *CXCL11*, *IFIT2*, *IFIT3,* and *APOBEC1*) were upregulated at 4 and 16 dpi in infected immunocompetent hamsters (Figure 8e,f, Figures S7 and S8).

Transcriptomes in SARS-CoV-2-infected immunocompetent hamsters and uninfected controls at 4 dpi were subjected to Ingenuity Pathway Analysis (IPA). The biological processes affected included the upregulation of host cell death, cytotoxicity, antimicrobial response, various types of immune cell development, recruitment and activation, and the inflammatory response. In contrast, biological functions associated with organismal survival were dampened in these animals (Table S1). At 16 dpi, the biological processes related to immune cell infiltration, edema, tissue necrosis, and organismal death were upregulated. At the same time, other functions related to lipid metabolism, including fatty acid metabolism and lipid transport, as well as the survival of organisms, were dampened in the infected immunocompetent hamsters compared to uninfected controls (Table S2). In contrast, the analysis of immunocompromised hamster lung transcriptome indicated that host biological functions associated with organismal survival, cell signaling, molecular transport, cell-mediated immunity, immune cell trafficking, and function were dampened, while cell death-related functions, such as apoptosis, were upregulated at 4 dpi (Table S3). However, some of these functions, including cell movement and molecular transport, and cell function and maintenance, were upregulated in the infected immunocompromised hamster lungs at 16 dpi. Importantly, humoral immune response functions, such as the number of B lymphocytes and quantity of immunoglobulin (Ig), were significantly downregulated in these animals (Table S4). This observation is consistent with the loss of antibody response in the infected immunocompromised animals. Together, the biological functions that are significantly perturbed in the immunocompetent and immunocompromised hamster lungs upon SARS-CoV-2 infection are consistent with and support the disease pathology and immune/antibody response in the respective animals.

### 3.12. Dampened Proinflammatory Response Pathways in the Lungs of SARS-CoV-2-Infected Immunocompromised Hamsters

**Figure 9:**
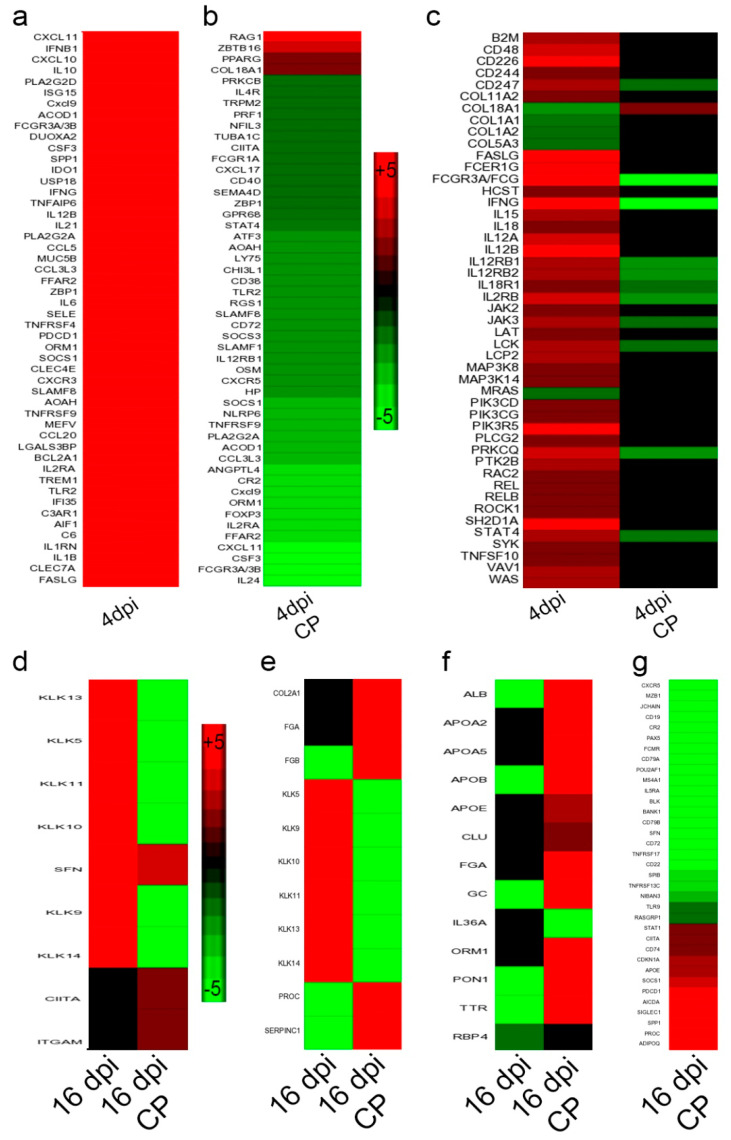
Expression of network genes in immunocompetent and immunocompromised hamsters infected with SARS-CoV-2. (**a**) Heat map of SDEG involved in the inflammatory response in immunocompetent hamster lungs at 4 dpi. (**b**) Heat map of SDEG involved in the inflammatory response in immunocompromised hamster lungs at 4 dpi (4 dpi CP). (**c**) Heat map of SDEG involved in NK cell activation network in immunocompetent (4 dpi) and immunocompromised (4 dpi CP) hamster lungs at 4 dpi. (**d**) Heat map of SDEG involved in canonical MPN-RON signaling in macrophages network in immunocompetent (16 dpi) and immunocompromised (16 dpi CP) hamster lungs at 16 dpi. (**e**) Heat map of SDEG involved in prothrombin signaling network in immunocompetent (16 dpi) and immunocompromised (16 dpi CP) hamster lungs at 16 dpi. (**f**) Heat map of SDEG involved in canonical LXR/RXR signaling pathway in immunocompetent (16 dpi) and immunocompromised (16 dpi CP) hamster lungs at 16 dpi. (**g**) Heat map of SDEG involved in B cell recruitment and accumulation network in immunocompetent (16 dpi) and immunocompromised (16 dpi CP) hamster lungs at 16 dpi. Scale bar shows up (red) and downregulation (green).

To determine the differential regulation of gene networks and pathways in the lungs of immunocompromised versus immunocompetent hamsters infected with SARS-CoV-2, we interrogated the RNAseq data at 4 and 16 dpi between these two groups after normalization to uninfected controls. The dampening of proinflammatory, innate immune function pathways, including phagosome formation, production of reactive oxygen and nitrogen species (ROS and RNS), dendritic cell maturation, and NK cell activation was noted at 4 dpi in the immunocompromised, compared to immunocompetent animals (Figure 9a–c and Figure S9). At 16 dpi, network genes involved in MPN-RON signaling and prothrombin signaling were upregulated in the SARS-CoV-2-infected immunocompetent hamsters (Figure 9d,e and Figure S10). In contrast, LXR/RXR signaling was upregulated in the infected immunocompromised hamsters (Figure 9f and Figure S10). However, the B cell receptor signaling and the network genes that determine the number of B cells were downregulated in this group (Figure 9g). This observation is consistent with and supported by the lack of antibodies in the sera of infected immunocompromised hamsters.

## 4. Discussion

The differential pathogenesis of COVID-19 between immunocompromised and immunocompetent individuals is poorly understood [24]. Similarly, predictors of disease severity, including the pathological manifestations of extrapulmonary organs upon SARS-CoV-2 infection in immunocompetent and immunocompromised hosts, are not well described. Previous studies have shown that Golden Syrian hamsters are reliable and predictable animal models that can recapitulate the pulmonary pathological manifestations of COVID-19 as seen in humans [25,26,27,28]. These reports show that following intranasal SARS-CoV-2 inoculation, high viral load and disease pathology were mainly localized to the trachea and lungs, with interstitial pneumonia being the main hallmark of severe disease [25,26,27,28].

Here, we report the dynamic changes in SARS-CoV-2 replication and pathological manifestations in pulmonary and extrapulmonary organs of immunocompetent and immunocompromised hamsters. We also report the molecular correlates of the host response to infection with different initial inoculum doses of SARS-CoV-2 in immunocompetent hamsters. Together, our data suggest that the histopathological manifestations caused by progressive SARS-CoV-2 infection predict COVID-19 severity better than individual measures of viral load, antibody response, and cytokine storm at the systemic or local (lungs) levels in the immunocompetent and immunocompromised hosts.

In patients with COVID-19, the highest viral load has been reported in the lungs, and low levels of viral RNA were detected in the cardiovascular, endocrine, gastrointestinal, urogenital, hemopoietic, and central nervous systems [29,30]. Furthermore, the infectious/live viruses were isolatable from most of the COVID-19 patients within 10 days of symptoms onset, despite detectable viral RNA for a longer time [31]. Consistent with these reports, we observed the highest live viral load in the pulmonary system of SARS-CoV-2-infected hamsters and a lower viral load was detected in the extrapulmonary organs. Notably, a similar viral load was observed in various tissues of LD and HD-infected hamsters, despite a 5-log difference in the inoculum used for infection between these two groups. In both LD and HD groups, infectious/live viruses were observed in the lungs up to 7 dpi; though the SARS-CoV-2 N gene transcript, an indicator of total viral burden (i.e., live and non-replicating viruses), was detected until 16 dpi. These findings are consistent with and supported by a previous study on the hamster model by Imai et al. [25]. Together, these observations suggest that the kinetics of viral replication and persistence in hamster tissues recapitulates the findings in COVID-19 cases.

In humans, the neutralizing antibodies generated following exposure to SARS-CoV-2 or vaccination constitute a significant determinant of virus clearance and protection [13,25]. Most of the SARS-CoV-2-infected individuals develop antiviral antibodies by 7–14 days [26]. In addition, clinical data shows that the high viral load and the severe disease correlate with increased antibody titer among COVID-19 cases [25,27]. Consistently, in our hamster studies, neutralizing antibodies were observed at 7 dpi, and virus clearance coincided with the appearance of these serum neutralizing antibodies. The virus-neutralizing antibody titer in these hamsters was proportional to the initial infectious dose of the virus, as reported in other studies [26,32,33]. Importantly, we did not observe any SARS-CoV-2 neutralizing antibodies in the sera of infected immunocompromised hamsters, which was also reported in a previous study [34]. Consequently, no virus clearance was observed in the tissues of SARS-CoV-2-infected immunocompromised animals up to 16 dpi. Interestingly, the lung transcriptome analysis at 16 dpi revealed significant downregulation of the B cell activation network genes, including CD19, CD22, CD72, FcgR, and IL-4 in the immunocompromised hamsters infected with SARS-CoV-2. It was reported that the Cyclophosphamide treatment could inhibit B cell activation, proliferation, differentiation, and immunoglobulin secretion in patients undergoing Cyclophosphamide therapy [17,35]. Indeed, patients treated with Cyclophosphamide for eosinophilic granulomatosis and polyangiitis did not produce any SARS-CoV-2-specific antibodies after they acquired symptomatic COVID-19 [36].

It is likely that the immunosuppression treatment of hamsters with Cyclophosphamide treatment would likely have diminished the number of T and B cells and/or abolished the ability to produce antibodies upon antigen exposure [36]. Furthermore, severely atrophied splenic lymphoid follicles were noted in the immunocompromised compared to immunocompetent hamsters. Thus, the lack of IL4-mediated B and/or T cell activation and, therefore, the antibody production during SARS-CoV-2 infection might have compromised the onset of an effective antiviral response in the immunosuppressed host.

A pathologic hallmark of severe COVID-19 cases is the onset of inflammatory “cytokine storm”, marked by elevated IL1B, TNFA, CCL2, IL6, MIP1A, and IL10 in the plasma [37]. Severe COVID-19 was also correlated with body weight loss [38]. Consistent with these reports, we observed elevated inflammatory cytokine levels in the infected immunocompetent and immunocompromised hamsters. Furthermore, body weight loss was significantly higher among HD-infected animals, which also showed more robust inflammation and disease pathology than LD-infected animals. However, the cytokine expression between LD and HD was not significantly different except for IFNG (4 dpi) and IL4 (16 dpi). Thus, it appears that once the active disease is established at about 4 dpi, the expression pattern of many of the cytokine storm molecules does not correlate with the infectious dose or the degree of pathological manifestations in the SARS-CoV-2-infected hamsters. In contrast, the SARS-CoV-2-infected immunocompromised hamsters had mild pulmonary lymphocytic infiltration and poor resolution of pneumonia and sustained a prolonged weight loss, compared to immunocompetent hamsters, which is consistent with previous observations in hamsters [25,34]. The elevated IL-10 expression in these hamsters might have played a role in reducing the immune cell infiltration to the site of infection (i.e., the lungs).

The lungs of immunocompetent hamsters infected with SARS-CoV-2 revealed upregulation of innate immune and inflammatory pathway genes, including IFNB and IFNL, NK cell activation, and hypercytokinemia as observed in previous studies [39]. A similar hyper induction of proinflammatory cytokines, such as IL6, IL1B, and IFNG, was observed in COVID-19 patients, and the level of induction in these cases was proportional to the severity of the disease [37]. Importantly, the induction of these inflammatory pathways persists even in the absence of active viral replication in the lungs, which is consistent with and supported by a recent multi-omics study performed in hamsters [40]. However, many of these pathways were dampened in the immunocompromised SARS-CoV-2-infected hamsters. The lack of optimal activation of proinflammatory immune response correlated with prolonged viral persistence in the infected immunocompromised hamsters. In addition, Cyclophosphamide treatment has been shown to inhibit Treg cell functions, and low dose Cyclophosphamide treatment in mice was reported to cause alterations in immune cells in the spleen and lymph nodes [39,41,42]. However, the causal link between Cyclophosphamide treatment and reduced proinflammatory cytokines and inflammation levels in COVID-19 cases remains unknown. Since the treatment regimen for COVID-19 patients includes a combination of anti-inflammatory agents, such as steroids, it is challenging to determine the differential immunomodulatory effect due to the treatment versus SARS-CoV-2 infection.

Clinical studies show that a significant proportion of COVID-19 patients had bronchial/bronchiolar wall thickening in computed tomography (CT) scan reports [43,44]. In general, the thickening of bronchiolar smooth muscles is a pathognomonic characteristic of asthma, which is mediated by a TH2 response, marked by elevated levels of IL4 and airway inflammation [45]. Further, CT scans from COVID-19 patients showed a thickening of blood vessels in the lungs, rupture of pararenal aortic aneurism, and cerebral aneurysm [46,47]. Although rhinoviruses have been reported to exacerbate asthma in infected patients [48,49], whether asthma is induced or exacerbated in COVID-19 patients remains unclear. We observed the thickening of bronchiolar smooth muscles in SARS-CoV-2-infected hamsters in an infectious-inoculum dose-dependent manner. In addition, as reported in human clinical studies [50], we observed extensive smooth muscle hypertrophy/hyperplasia in the SARS-CoV-2-infected hamster pulmonary arterioles and renal arterioles and the rupture of pulmonary vessels in some animals. While the exact mechanism underlying vascular thickening during SARS-CoV-2 infection is not fully understood, the disruption of the renin-angiotensin system during COVID-19 could contribute to this anomaly [51].

Autopsies have revealed that cortical degeneration and necrosis, adrenalitis, and cortical hyperplasia of adrenal glands are associated with COVID-19 infection [52,53]. Similar adrenal cortical and medullary lesions were also observed in the hamsters, affecting deregulating blood pressure during SARS-CoV-2 infection [54,55]. However, the causal association between adrenal cortical/medullary insufficiency and the severity of COVID-19 is yet to be unraveled. Clinical studies have also shown microvesicular hepatic steatosis among COVID-19 cases with or without underlying health conditions [56,57]. Although previous studies in the hamster model did not report any abnormality in the liver [16,17], we observed mild degeneration and steatosis in both LD and HD SARS-CoV-2- infected hamsters. The discrepancy between these studies could be due to the inherent differences in the experimental design and the extent of pathological analysis performed.

Severe COVID-19 is associated with acute kidney injury and renal failure [58,59,60]. Autopsy reports indicate the presence of proximal convoluted tubular epithelial necrosis, loss of brush border, and detachment into the lumen of the kidney in patients who died of COVID-19 and during biopsy [58,59]. Additional symptoms of acute kidney injury, including proteinuria and elevated serum creatinine levels, were also noticed in these cases [59,60]. Consistent with these reports, we observed acute tubular epithelial necrosis of kidneys following SARS-CoV-2 infection in immunocompetent and immunocompromised hamsters. However, the precise mechanism of renal injury during COVID-19 is yet to be determined.

## 5. Conclusions

**Figure 10:**
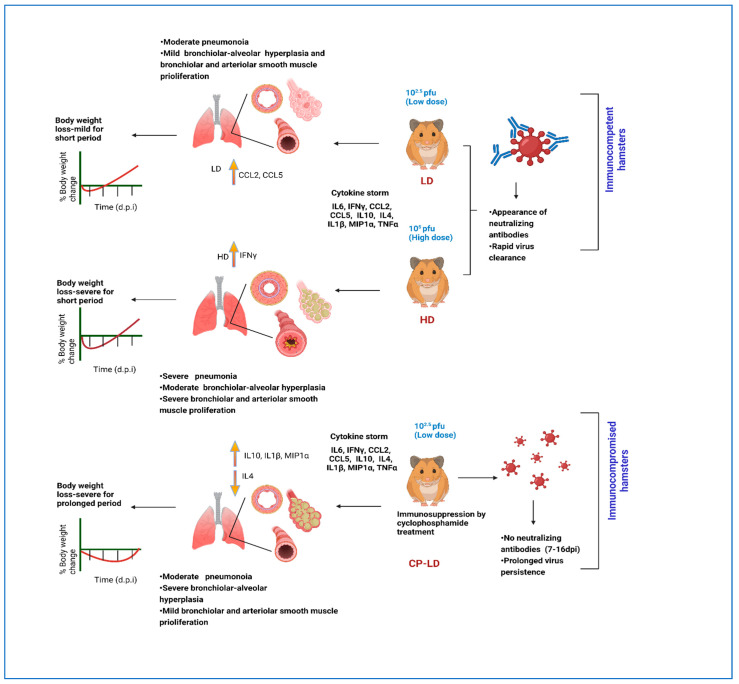
Graphical summary of experimental design and key findings of this study. LD infection of immunocompetent hamsters was associated with mild body weight changes, rapid viral clearance, elevated CCL2 and CCL5 expression, and the presence of neutralizing plasma antibodies. HD infection of immunocompetent hamsters was associated with severe body weight loss and disease pathology, elevated IFN-γ expression, and strong neutralizing antibody in the plasma. In contrast, LD infection of immunocompromised hamsters was associated with less severe but prolonged bodyweight changes, persistent viral load, elevated IL10, IL1b, MIP1A, and dampened IL4 expression. No neutralizing antibodies were detected in the plasma of these animals. LD—low-dose infection; HD—high-dose infection; CP-LD—Cyclophosphamide (immunosuppressed)-treated-low dose infection.

In conclusion, our findings reveal the kinetics of viral replication, antibody response, and associated disease pathology of various internal organs in immunocompetent and immunocompromised hamsters following SARS-CoV-2 infection (Figure 10). The histopathologic findings in our hamster models closely mimic the clinical and pathological manifestations observed in human COVID-19 cases. The two hamster models described in this work could be used to unravel the pathogenesis, tissue injury, and viral transmission within and outside of the infected host. These models can also serve as a preclinical tool to evaluate potential intervention strategies such as therapeutics and vaccines to combat the ongoing COVID-19 pandemic.

## Figures and Tables

**Table 1:** The detectable SARS-CoV-2 in infected hamster organs at different time points.

	Day 2			Day 4			Day 7			Day 12			Day 16		
Organ	LD	HD	CP-LD	LD	HD	CP-LD	LD	HD	CP-LD	LD	HD	CP-LD	LD	HD	CP-LD
Nasal wash	×	×	×	×	×	×	×		×	-	-	×	-	-	×
Nasal turbinates	×	×	×	×	×	×	×	×	×	-	-	×	-	-	×
Larynx and trachea	×	×	×	×	×	×			×	-	-	×	-	-	×
Lung	×	×	×	×	×	×	×	×	×	-	-	×	-	-	×
Heart	×	×	×	×	×	×	-	-	×	-	-	×	-	-	×
Adrenal	×	×	×	×	×	×	-	-		-	-	-	-	-	×
Kidney	×	×	×			×	-	-	×	-	-	-	-	-	-
Epididymal fat	×	×	×	×	×	×	-	-	×	-	-	-	-	-	×
Brain with olfactory bulb	×	×	×	×	×	×			×						×
Eye	×		×	×		×			×			×			×
Colon	×		×		×										
Bone marrow	×		×	×	×	×			×						×
Liver		×	×	×		×									
Spleen		×	×	×	×	×			×						

LD—low-dose, HD—high-dose, CP-LD—Cyclophosphamide-treated (immunosuppressed) and low-dose infected, ×—detectable viral load in respective organs. —no viral particles detected.

**Table 2:** Histopathological scoring of SARS-CoV-2-infected hamster lungs.

Lesion Parameters (Scale)	Virus Dose/Treatment	Day 4 (Mean ± SD)	Day 7 (Mean ± SD)	Day 16 (Mean ± SD)
Mononuclear cells infiltration (0–5)	LD	2.33 ± 0.577	3.667 ± 0.288	2 ± 0
	HD	3.33 ± 0.577	4.33 ± 1.15	4 ± 0
	CP-LD	2 ± 0	2.33 ± 0.288	2 ± 0
Alveolar hyperplasia (0–5)	LD	0.66 ± 0.577	1.833 ± 1.25	1.16 ± 0.76
	HD	2.66 ± 0.57	5 ± 0	4 ± 0
	CP-LD	2.16 ± 0.288	2 ± 0	3 ± 0
Interstitial and alveolar edema (0–4)	LD	0.66 ± 0.288	2.33 ± 0.577	0.166 ± 0.288
	HD	1.33 ± 0.577	1.66 ± 0.577	0.5 ± 0.7
	CP-LD	0.66 ± 0.288	2.166 ± 0.288	0
Emphysema (0–4)	LD	0.33 ± 0.577	1 ± 0	0
	HD	1.33 ± 1.15	2.66 ± 0.577	0.5 ± 0.707
	CP-LD	1.66 ± 0.577	1 ± 0	1 ± 0
Bronchiolar epithelial hyperplasia and inflammation (0–4)	LD	1.66 ± 0.577	2.66 ± 0.577	1.83 ± 0.28
	HD	2.66 ± 1.15	4 ± 0	3.5 ± 0.7
	CP-LD	2.33 ± 0.577	2 ± 0	2.33 ± 1.52
Bronchiolar smooth muscle hyperplasia (0–4)	LD	0.5 ± 0.866	0.66 ± 1.15	0.66 ± 1.15
	HD	0.66 ± 1.15	1 ± 1	3 ± 0
	CP-LD	1 ± 1	0	0
Vascular lesions; vasculitis, congestion, perivasculitis (0–4)	LD	2 ± 0	2.66 ± 0.577	1.33 ± 0.577
	HD	2.66 ± 0.577	3.66 ± 0.577	2 ± 0
	CP-LD	1.33 ± 0.577	1.66 ± 1.15	1.5 ± 0.70
Hyperplasia/hypertrophy of vascular smooth muscle (0–4)	LD	1.33 ± 1.15	1 ± 0.866	2 ± 1
	HD	2.83 ± 0.76	2.16 ± 1.04	4 ± 0
	CP-LD	1 ± 1	1.66 ± 0.577	3 ± 0
Foamy macrophages (0–4)	LD	0 ± 0	2 ± 0	1 ± 0
	HD	0 ± 0	1.66 ± 0.577	0.5 ± 0.7
	CP-LD	0 ± 0	1 ± 0	0.5 ± 0.866

LD—low-dose, HD—high-dose, CP-LD—Cyclophosphamide-treated (immunosuppressed) and low-dose infected.

## Data Availability

The RNAseq data have been submitted to Gene Expression Omnibus (GEO) of the NCBI (accession numberSUB10759538). The gene expression data of the network/pathway presented in this article are available in the Supplementary Materials Section. Other experimental datasets used in this manuscript are available upon request to the communication author; these data are not publically available due to ethical reasons from the funding agency.

## Supplementary Materials

The following supporting information can be downloaded at: https://www.mdpi.com/article/10.3390/biomedicines10061343/s1, Figure S1: Histopathology of the trachea in SARS-CoV-2-infected hamsters. Figure S2: qPCR analysis of B and T cell lineage markers in LD and CP-LD-infected hamster lungs. Figure S3: Histopathology of the adrenal gland in SARS-CoV-2-infected hamsters. Figure S4: White pulp content in the spleen of SARS-CoV-2-infected hamsters. Figure S5: Acute renal tubular necrosis in SARS-CoV-2-infected hamsters. Figure S6: Top canonical pathways operative in immunocompetent hamster lungs infected with low-dose SARS-CoV-2 at 4 days post-infection. Figure S7: Top canonical pathways operative in immunocompetent hamster lungs infected with low-dose SARS-CoV-2 at 16 days post-infection. Figure S8: Canonical hypercytokinemia/hyperchemokinemia pathway map. Figure S9: Canonical interferon signaling pathway map. Figure S10: Top canonical pathways operative in immunocompromised hamster lungs infected with low-dose SARS-CoV-2 at 4 days post-infection. Figure S11: Top canonical pathways operative in immunocompromised hamster lungs infected with low-dose SARS-CoV-2 at 16 days post-infection. Table S1: Differentially regulated biological functions at 4 dpi versus uninfected hamster lungs. Table S2: Differentially regulated biological functions at 16 dpi versus uninfected hamster lungs. Table S3: Differentially regulated biological functions in immunosuppressed SARS-CoV-2-infected hamster lungs at 4 dpi. Table S4: Differentially regulated biological functions in immunosuppressed SARS-CoV-2-infected hamster lungs at 16 dpi. Table S5: List of primers used for hamster and SARS-CoV-2 genes in the present study.

## References

[1] WHO Coronavirus (COVID-19) Dashboard. https://covid19.who.int/.

[2] Qin C., Zhou L., Hu Z., Zhang S., Yang S., Tao Y., Xie C., Ma K., Shang K., Wang W. (2020). Dysregulation of Immune Response in Patients with Coronavirus 2019 (COVID-19) in Wuhan, China. Clin. Infect. Dis..

[3] Hadjadj J., Yatim N., Barnabei L., Corneau A., Boussier J., Smith N., Pere H., Charbit B., Bondet V., Chenevier-Gobeaux C. (2020). Impaired type I interferon activity and inflammatory responses in severe COVID-19 patients. Science.

[4] Zhou F., Yu T., Du R., Fan G., Liu Y., Liu Z., Xiang J., Wang Y., Song B., Gu X. (2020). Clinical course and risk factors for mortality of adult inpatients with COVID-19 in Wuhan, China: A retrospective cohort study. Lancet.

[5] Chang M.G., Yuan X., Tao Y., Peng X., Wang F.S., Xie L., Sharma L., Dela Cruz C.S., Qin E. (2020). Time Kinetics of Viral Clearance and Resolution of Symptoms in Novel Coronavirus Infection. Am. J. Respir. Crit. Care Med..

[6] Wei J., Zhao J., Han M., Meng F., Zhou J. (2020). SARS-CoV-2 infection in immunocompromised patients: Humoral versus cell-mediated immunity. J. Immunother. Cancer.

[7] Lang M., Som A., Carey D., Reid N., Mendoza D.P., Flores E.J., Li M.D., Shepard J.O., Little B.P. (2020). Pulmonary Vascular Manifestations of COVID-19 Pneumonia. Radiol. Cardiothorac. Imaging.

[8] Roberts C.M., Levi M., McKee M., Schilling R., Lim W.S., Grocott M.P.W. (2020). COVID-19: A complex multisystem disorder. Br. J. Anaesth..

[9] Lacy J.M., Brooks E.G., Akers J., Armstrong D., Decker L., Gonzalez A., Humphrey W., Mayer R., Miller M., Perez C. (2020). COVID-19: Postmortem Diagnostic and Biosafety Considerations. Am. J. Forensic. Med. Pathol..

[10] Carsana L., Sonzogni A., Nasr A., Rossi R.S., Pellegrinelli A., Zerbi P., Rech R., Colombo R., Antinori S., Corbellino M. (2020). Pulmonary post-mortem findings in a series of COVID-19 cases from northern Italy: A two-centre descriptive study. Lancet Infect. Dis..

[11] Elsoukkary S.S., Mostyka M., Dillard A., Berman D.R., Ma L.X., Chadburn A., Yantiss R.K., Jessurun J., Seshan S.V., Borczuk A.C. (2021). Autopsy Findings in 32 Patients with COVID-19: A Single-Institution Experience. Pathobiology.

[12] Falasca L., Nardacci R., Colombo D., Lalle E., Di Caro A., Nicastri E., Antinori A., Petrosillo N., Marchioni L., Biava G. (2020). Postmortem Findings in Italian Patients with COVID-19: A Descriptive Full Autopsy Study of Cases with and without Comorbidities. J. Infect. Dis..

[13] Khoury D.S., Cromer D., Reynaldi A., Schlub T.E., Wheatley A.K., Juno J.A., Subbarao K., Kent S.J., Triccas J.A., Davenport M.P. (2021). Neutralizing antibody levels are highly predictive of immune protection from symptomatic SARS-CoV-2 infection. Nat. Med..

[14] Case J.B., Bailey A.L., Kim A.S., Chen R.E., Diamond M.S. (2020). Growth, detection, quantification, and inactivation of SARS-CoV-2. Virology.

[15] Matsuyama S., Nao N., Shirato K., Kawase M., Saito S., Takayama I., Nagata N., Sekizuka T., Katoh H., Kato F. (2020). Enhanced isolation of SARS-CoV-2 by TMPRSS2-expressing cells. Proc. Natl. Acad. Sci. USA.

[16] Mendoza E.J., Manguiat K., Wood H., Drebot M. (2020). Two Detailed Plaque Assay Protocols for the Quantification of Infectious SARS-CoV-2. Curr. Protoc. Microbiol..

[17] Schaecher S.R., Stabenow J., Oberle C., Schriewer J., Buller R.M., Sagartz J.E., Pekosz A. (2008). An immunosuppressed Syrian golden hamster model for SARS-CoV infection. Virology.

[18] Subbian S., Tsenova L., Holloway J., Peixoto B., O’Brien P., Dartois V., Khetani V., Zeldis J.B., Kaplan G. (2016). Adjunctive Phosphodiesterase-4 Inhibitor Therapy Improves Antibiotic Response to Pulmonary Tuberculosis in a Rabbit Model. EBioMedicine.

[19] Kumar R., Subbian S. (2021). Immune Correlates of Non-Necrotic and Necrotic Granulomas in Pulmonary Tuberculosis: A Pilot Study. J. Respir..

[20] Perera R., Ko R., Tsang O.T.Y., Hui D.S.C., Kwan M.Y.M., Brackman C.J., To E.M.W., Yen H.L., Leung K., Cheng S.M.S. (2021). Evaluation of a SARS-CoV-2 Surrogate Virus Neutralization Test for Detection of Antibody in Human, Canine, Cat, and Hamster Sera. J. Clin. Microbiol..

[21] Subbian S., Bandyopadhyay N., Tsenova L., O’Brien P., Khetani V., Kushner N.L., Peixoto B., Soteropoulos P., Bader J.S., Karakousis P.C. (2013). Early innate immunity determines outcome of Mycobacterium tuberculosis pulmonary infection in rabbits. Cell Commun. Signal..

[22] Subbian S., O’Brien P., Kushner N.L., Yang G., Tsenova L., Peixoto B., Bandyopadhyay N., Bader J.S., Karakousis P.C., Fallows D. (2013). Molecular immunologic correlates of spontaneous latency in a rabbit model of pulmonary tuberculosis. Cell Commun. Signal..

[23] Karwaciak I., Salkowska A., Karas K., Dastych J., Ratajewski M. (2021). Nucleocapsid and Spike Proteins of the Coronavirus SARS-CoV-2 Induce IL6 in Monocytes and Macrophages-Potential Implications for Cytokine Storm Syndrome. Vaccines.

[24] Suess C., Hausmann R. (2020). Gross and histopathological pulmonary findings in a COVID-19 associated death during self-isolation. Int. J. Leg. Med..

[25] Imai M., Iwatsuki-Horimoto K., Hatta M., Loeber S., Halfmann P.J., Nakajima N., Watanabe T., Ujie M., Takahashi K., Ito M. (2020). Syrian hamsters as a small animal model for SARS-CoV-2 infection and countermeasure development. Proc. Natl. Acad. Sci. USA.

[26] Sia S.F., Yan L.M., Chin A.W.H., Fung K., Choy K.T., Wong A.Y.L., Kaewpreedee P., Perera R., Poon L.L.M., Nicholls J.M. (2020). Pathogenesis and transmission of SARS-CoV-2 in golden hamsters. Nature.

[27] Rosenke K., Meade-White K., Letko M., Clancy C., Hansen F., Liu Y., Okumura A., Tang-Huau T.L., Li R., Saturday G. (2020). Defining the Syrian hamster as a highly susceptible preclinical model for SARS-CoV-2 infection. Emerg. Microbes Infect..

[28] Chan J.F., Zhang A.J., Yuan S., Poon V.K., Chan C.C., Lee A.C., Chan W.M., Fan Z., Tsoi H.W., Wen L. (2020). Simulation of the Clinical and Pathological Manifestations of Coronavirus Disease 2019 (COVID-19) in a Golden Syrian Hamster Model: Implications for Disease Pathogenesis and Transmissibility. Clin. Infect. Dis..

[29] Deinhardt-Emmer S., Wittschieber D., Sanft J., Kleemann S., Elschner S., Haupt K.F., Vau V., Haring C., Rodel J., Henke A. (2021). Early postmortem mapping of SARS-CoV-2 RNA in patients with COVID-19 and the correlation with tissue damage. eLife.

[30] Dong M., Zhang J., Ma X., Tan J., Chen L., Liu S., Xin Y., Zhuang L. (2020). ACE2, TMPRSS2 distribution and extrapulmonary organ injury in patients with COVID-19. Biomed. Pharmacother..

[31] Wolfel R., Corman V.M., Guggemos W., Seilmaier M., Zange S., Muller M.A., Niemeyer D., Jones T.C., Vollmar P., Rothe C. (2020). Virological assessment of hospitalized patients with COVID-2019. Nature.

[32] Tostanoski L.H., Wegmann F., Martinot A.J., Loos C., McMahan K., Mercado N.B., Yu J., Chan C.N., Bondoc S., Starke C.E. (2020). Ad26 vaccine protects against SARS-CoV-2 severe clinical disease in hamsters. Nat. Med..

[33] O’Donnell K.L., Pinski A.N., Clancy C.S., Gourdine T., Shifflett K., Fletcher P., Messaoudi I., Marzi A. (2021). Pathogenic and transcriptomic differences of emerging SARS-CoV-2 variants in the Syrian golden hamster model. EBioMedicine.

[34] Brocato R.L., Principe L.M., Kim R.K., Zeng X., Williams J.A., Liu Y., Li R., Smith J.M., Golden J.W., Gangemi D. (2020). Disruption of Adaptive Immunity Enhances Disease in SARS-CoV-2-Infected Syrian Hamsters. J. Virol..

[35] Zhu L.P., Cupps T.R., Whalen G., Fauci A.S. (1987). Selective effects of cyclophosphamide therapy on activation, proliferation, and differentiation of human B cells. J. Clin. Investig..

[36] Schramm M.A., Venhoff N., Wagner D., Thiel J., Huzly D., Craig-Mueller N., Panning M., Hengel H., Kern W.V., Voll R.E. (2020). COVID-19 in a Severely Immunosuppressed Patient with Life-Threatening Eosinophilic Granulomatosis with Polyangiitis. Front. Immunol..

[37] Ramasamy S., Subbian S. (2021). Critical Determinants of Cytokine Storm and Type I Interferon Response in COVID-19 Pathogenesis. Clin. Microbiol. Rev..

[38] Di Filippo L., De Lorenzo R., D’Amico M., Sofia V., Roveri L., Mele R., Saibene A., Rovere-Querini P., Conte C. (2021). COVID-19 is associated with clinically significant weight loss and risk of malnutrition, independent of hospitalisation: A post-hoc analysis of a prospective cohort study. Clin. Nutr..

[39] Nouailles G., Wyler E., Pennitz P., Postmus D., Vladimirova D., Kazmierski J., Pott F., Dietert K., Muelleder M., Farztdinov V. (2021). Temporal omics analysis in Syrian hamsters unravel cellular effector responses to moderate COVID-19. Nat. Commun..

[40] Cantwell A.M., Singh H., Platt M., Yu Y., Lin Y.H., Ikeno Y., Hubbard G., Xiang Y., Gonzalez-Juarbe N., Dube P.H. (2021). Kinetic Multi-omic Analysis of Responses to SARS-CoV-2 Infection in a Model of Severe COVID-19. J. Virol..

[41] Traverso I., Fenoglio D., Negrini S., Parodi A., Battaglia F., Kalli F., Conteduca G., Tardito S., Traverso P., Indiveri F. (2012). Cyclophosphamide inhibits the generation and function of CD8(+) regulatory T cells. Hum. Immunol..

[42] Lutsiak M.E., Semnani R.T., De Pascalis R., Kashmiri S.V., Schlom J., Sabzevari H. (2005). Inhibition of CD4(+)25+ T regulatory cell function implicated in enhanced immune response by low-dose cyclophosphamide. Blood.

[43] Motawea A.M., Omar S., Yasin R. (2021). Imaging of COVID-19 simulators. Egypt. J. Radiol. Nucl. Med..

[44] Ye Z., Zhang Y., Wang Y., Huang Z., Song B. (2020). Chest CT manifestations of new coronavirus disease 2019 (COVID-19): A pictorial review. Eur. Radiol..

[45] Ghosh S., Das S., Mondal R., Abdullah S., Sultana S., Singh S., Sehgal A., Behl T. (2021). A review on the effect of COVID-19 in type 2 asthma and its management. Int. Immunopharmacol..

[46] Rinaldi L.F., Marazzi G., Marone E.M. (2020). Endovascular Treatment of a Ruptured Pararenal Abdominal Aortic Aneurysm in a Patient with Coronavirus Disease-2019: Suggestions and Case Report. Ann. Vasc. Surg..

[47] Shih M., Swearingen B., Rhee R. (2020). Ruptured Abdominal Aortic Aneurysm Treated with Endovascular Repair in a Patient with Active COVID-19 Infection during the Pandemic. Ann. Vasc. Surg..

[48] Zheng X.Y., Xu Y.J., Guan W.J., Lin L.F. (2018). Regional, age and respiratory-secretion-specific prevalence of respiratory viruses associated with asthma exacerbation: A literature review. Arch. Virol..

[49] Morais-Almeida M., Bousquet J. (2020). COVID-19 and asthma: To have or not to have T2 inflammation makes a difference?. Pulmonology.

[50] Suzuki Y.J., Nikolaienko S.I., Dibrova V.A., Dibrova Y.V., Vasylyk V.M., Novikov M.Y., Shults N.V., Gychka S.G. (2021). SARS-CoV-2 spike protein-mediated cell signaling in lung vascular cells. Vasc. Pharmacol..

[51] Mehta P.K., Griendling K.K. (2007). Angiotensin II cell signaling: Physiological and pathological effects in the cardiovascular system. Am. J. Physiol. Cell Physiol..

[52] Freire Santana M., Borba M.G.S., Baia-da-Silva D.C., Val F., Alexandre M.A.A., Brito-Sousa J.D., Melo G.C., Queiroga M.V.O., Leao Farias M.E., Camilo C.C. (2020). Case Report: Adrenal Pathology Findings in Severe COVID-19: An Autopsy Study. Am. J. Trop. Med. Hyg..

[53] Mondello C., Roccuzzo S., Malfa O., Sapienza D., Gualniera P., Ventura Spagnolo E., Di Nunno N., Salerno M., Pomara C., Asmundo A. (2021). Pathological Findings in COVID-19 as a Tool to Define SARS-CoV-2 Pathogenesis. A Systematic Review. Front. Pharmacol..

[54] Kidambi S., Kotchen J.M., Grim C.E., Raff H., Mao J., Singh R.J., Kotchen T.A. (2007). Association of adrenal steroids with hypertension and the metabolic syndrome in blacks. Hypertension.

[55] Moneva M.H., Gomez-Sanchez C.E. (2002). Pathophysiology of adrenal hypertension. Semin. Nephrol..

[56] Ji D., Qin E., Xu J., Zhang D., Cheng G., Wang Y., Lau G. (2020). Non-alcoholic fatty liver diseases in patients with COVID-19: A retrospective study. J. Hepatol..

[57] Xu L., Liu J., Lu M., Yang D., Zheng X. (2020). Liver injury during highly pathogenic human coronavirus infections. Liver Int..

[58] Su H., Yang M., Wan C., Yi L.X., Tang F., Zhu H.Y., Yi F., Yang H.C., Fogo A.B., Nie X. (2020). Renal histopathological analysis of 26 postmortem findings of patients with COVID-19 in China. Kidney Int..

[59] Sharma P., Uppal N.N., Wanchoo R., Shah H.H., Yang Y., Parikh R., Khanin Y., Madireddy V., Larsen C.P., Jhaveri K.D. (2020). COVID-19-Associated Kidney Injury: A Case Series of Kidney Biopsy Findings. J. Am. Soc. Nephrol..

[60] Ahmadian E., HosseiniyanKhatibi S.M., RaziSoofiyani S., Abediazar S., Shoja M.M., Ardalan M., ZununiVahed S. (2021). COVID-19 and kidney injury: Pathophysiology and molecular mechanisms. Rev. Med. Virol..

