# Supplementary material for: Comprehensive Analysis of Disease Pathology in Immunocompetent and Immunocompromised Hosts following Pulmonary SARS-CoV-2 Infection
Source: Biomedicines. 2022 Jun 7;10(6):1343. doi: 10.3390/biomedicines10061343 (PMC9219777; doi:10.3390/biomedicines10061343)
Supplement: Supplementary file 1 [file biomedicines-10-01343-s001.zip › biomedicines-1707129-supplementary.pdf]

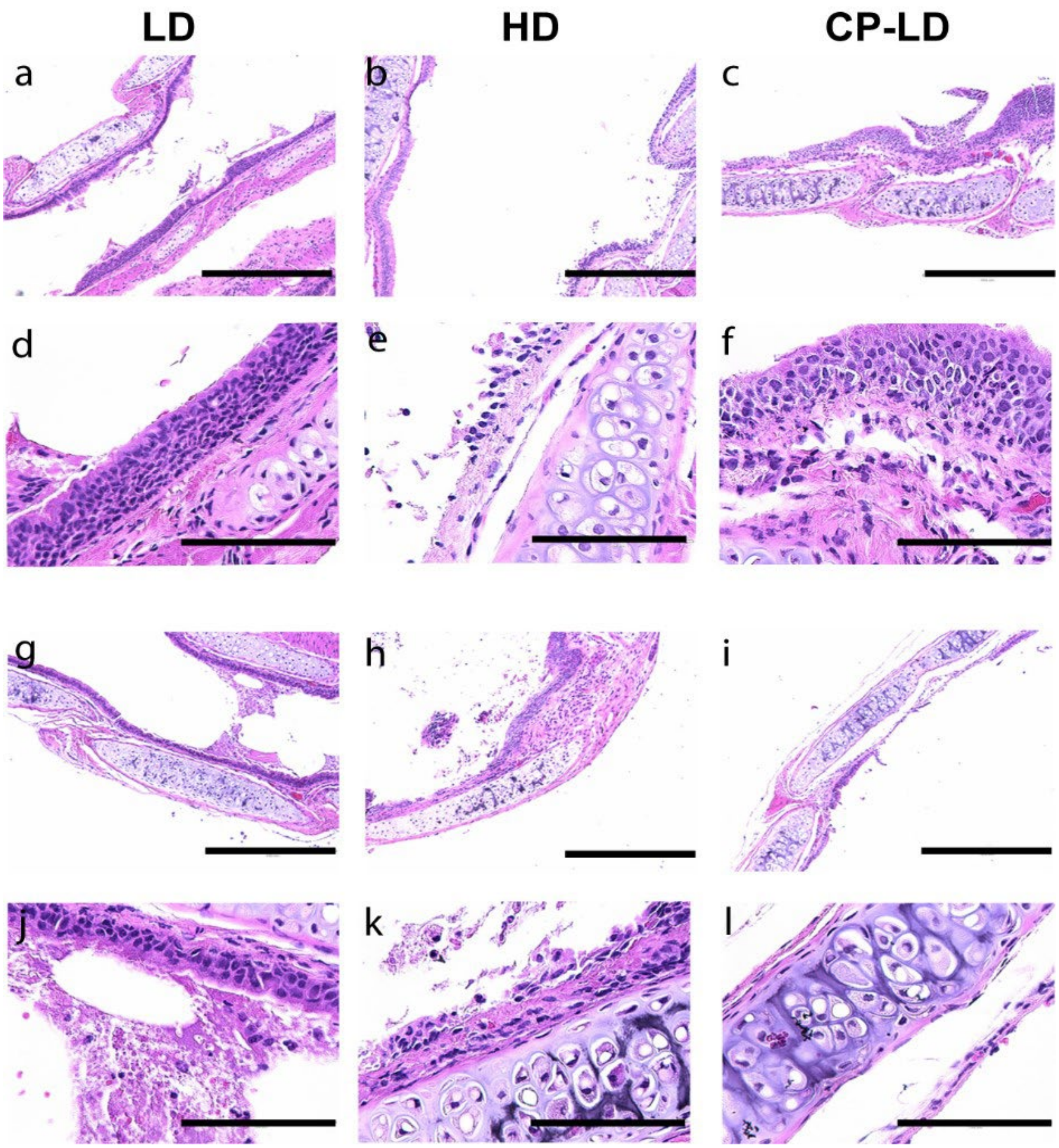

Figure S1. Histopathology of the trachea in SARS-CoV-2 infected hamsters.
Histopathological analysis of trachea (4, 7 and 16 dpi., n=3 per time point) revealed squamous metaplasia of tracheal epithelial cells **(a, d)** and accumulation of mucinous exudates and macrophages and neutrophils in the tracheal lumen **(g, j)** in LD infected hamsters at 7 dpi. Epithelial metaplasia **(b, h, k)** and mucosal detachment **(b, e)** in HD infected hamsters on 4 and 7 d.p.i. Severe stratified squamous metaplasia **(c, f)** at 4 dpi and detachment of epithelial cells **(i, l)** on 16 d.p.i. in the cyclophosphamide treated LD infected hamsters. The images **a-c, g-I** are 100x and **d-f, j-l** are 400x magnifications. Scale bar represents 100 µm (**d-f, j-l)** or 400 µm (**a-c, g-l)**. **LD**-low dose; **HD**-high dose; **CP-LD**-cyclophosphamide treated (immunosuppressed) and low dose infected groups.

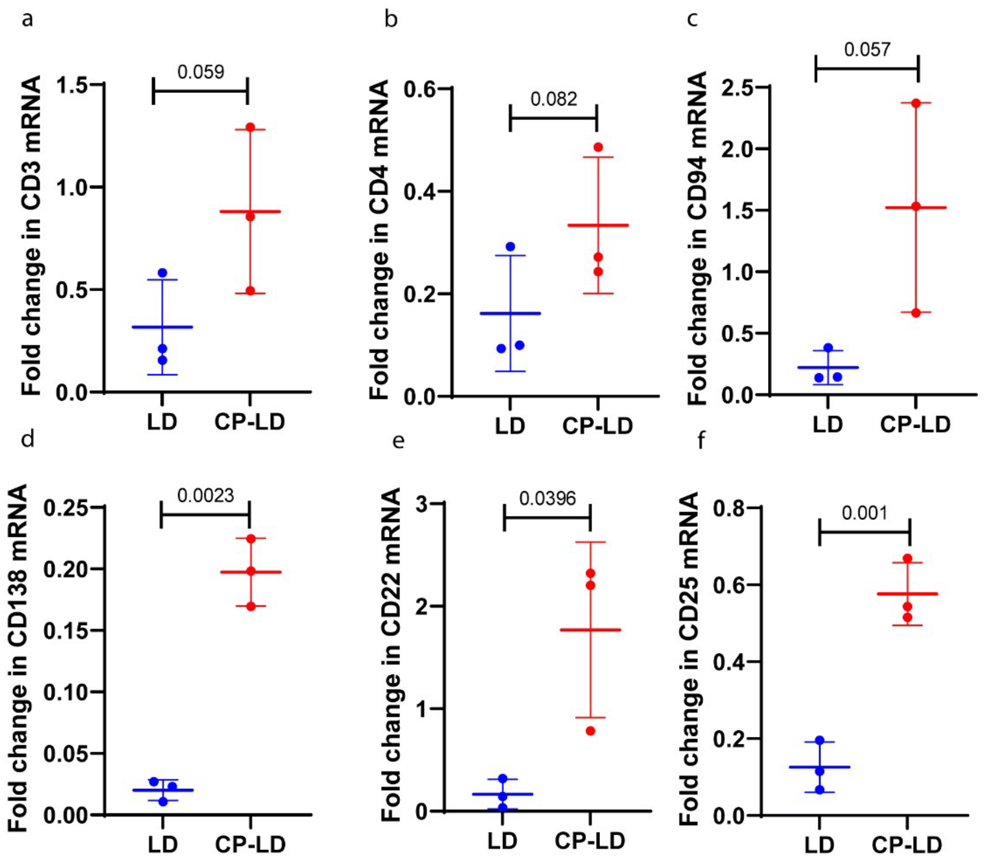

Figure S2: qPCR analysis of B and T cell lineage markers in LD and CP-LD infected hamster lungs. The level of T cell markers CD3, CD4, CD94 expressionin the lungs was not significantly different between LD and CP-LD infected animals at 7 dpi **(a, b, c)**, whereas expression of plasma cell marker CD138 **(d)**, B cell markerCD22 **(e)** and Treg marker CD25 **(f)** was significantly down-regulated in LD, compared to CP-LD infected hamster lungs. The values are normalized with GAPDH/b-actin transcripts and represent the fold change in expression compared to uninfected hamsters. Statistical analysis was performed by two-sided Welch's t-test. Data represent mean ± sd **(a-f).** The blue, green, and red indicate LD, HD, and CP-LD infected hamsters, respectively, and each dot represents an individual hamster data **(a-f)**. **LD**-low dose; **CP-LD**-cyclophosphamide treated (immunosuppressed) and low dose infected groups.

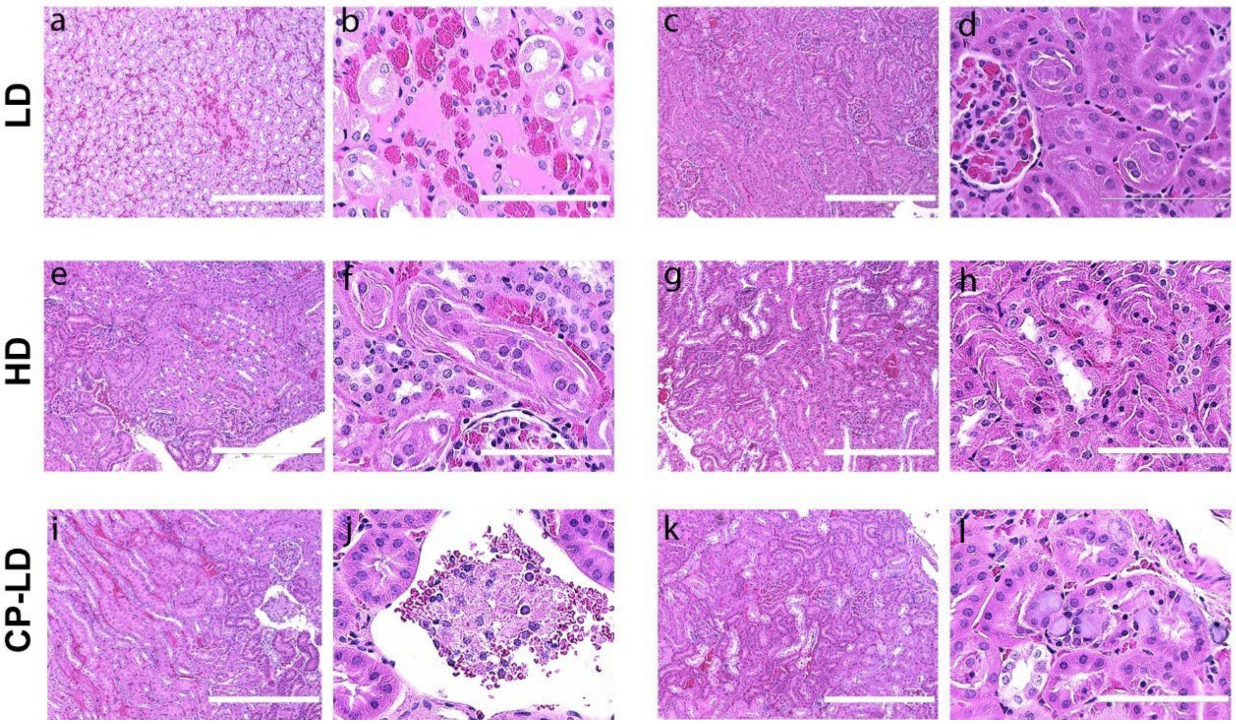

Figure S3. Histopathology of the adrenal gland in SARS-CoV-2 infected hamsters. A section of the adrenal cortex showing degenerated cells with pyknotic nuclei (**a, b**) on 4 dpi. and liquefaction necrosis in the medulla (**c, d**) on day 7 dpi. Hyperplasia/hypertrophy of adrenal cortex (**e, f**) and mild degeneration in the cortical cells (**f**) and liquefaction necrosis in the medullary chords (**g, h**) on 7 dpi. In CP-LD infected hamsters, severe degeneration and liquefaction necrosis in the cortex is seen as eosinophilic necrotic material (**i, j**) on 4 dpi and chromatolysis and necrosis of chromaffin cells in the medulla (**k, l**) on 7 dpi were observed. The images **a, e, i, c, g, k** are 100x and **b, f, j, d, h, l** are 400x magnifications. Scale bar represents 100 μm (**b, f, j, d, h, l**) or 400 μm (**a, e, i, c, g, k**). **LD**-low dose; **HD**-high dose; **CP-LD**-cyclophosphamide treated (immunosuppressed) and low dose infected groups.

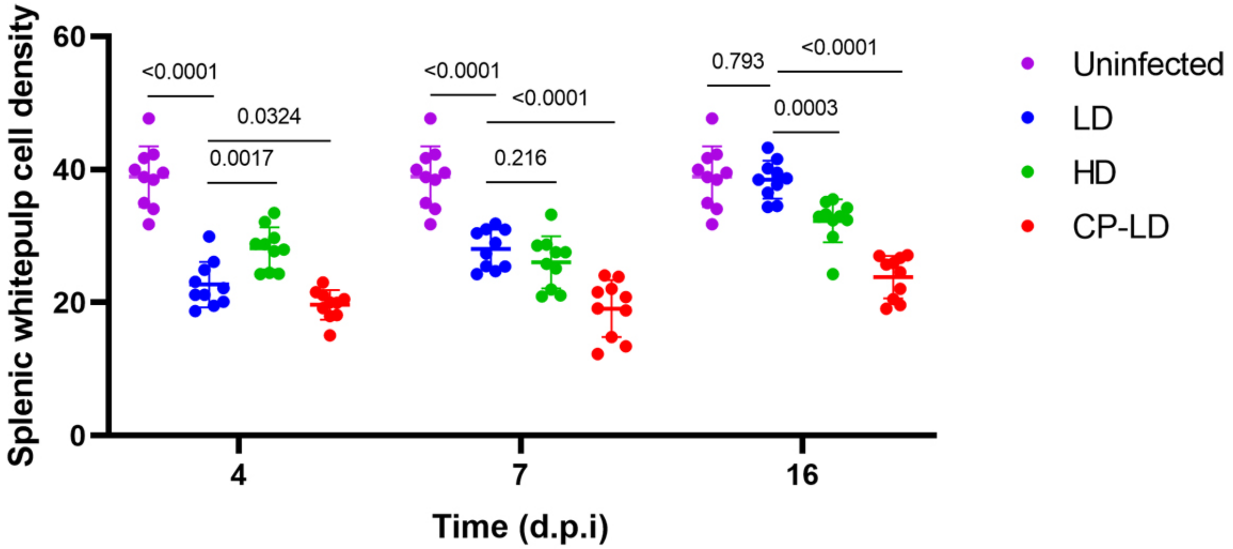

Figure S4. White pulp content in the spleen of SARS-CoV-2 infected hamsters. Histopathological analysis at 4, 7 and 16 dpi revealed differential white pulp density in the spleen upon SARS-CoV-2 infection. **LD**-low dose; **HD**-high dose; **CP-LD**-cyclophosphamide treated (immunosuppressed) and low dose infected groups.

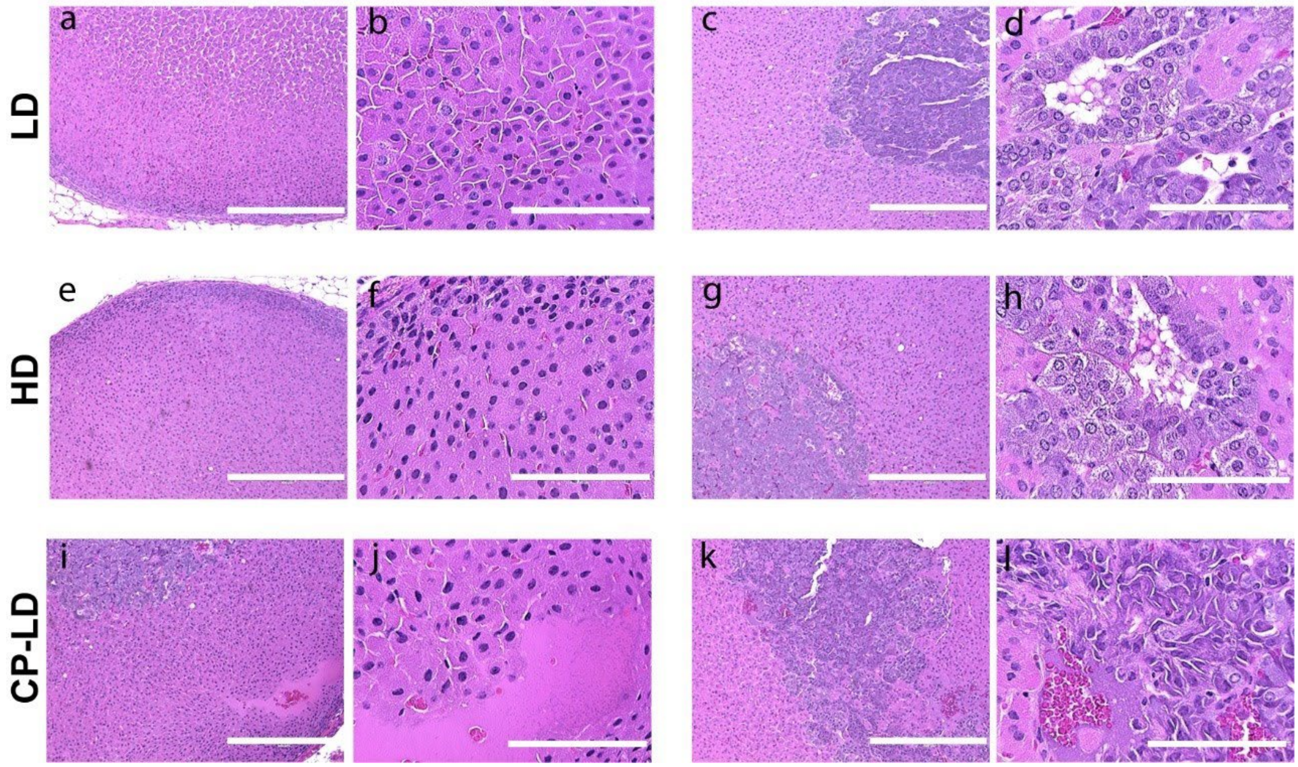

Figure S5. Acute renal tubular necrosis in SARS-CoV-2 infected hamsters. Histopathological analysis of kidney (4, 7 and 16 dpi. n=3 per time point) revealed that the severe necrosis of renal tubules with karyolysis and accumulation of eosinophilic material in the tubular lumen **(a, b)** and tubular degeneration and mild infiltration of lymphocytes in the interstitium **(c, d)** following LD infection (4 d.p.i). HD infected hamsters' kidneys showed severe acute tubular necrosis of proximal convoluted tubules with pyknosis and loss of brush border into the lumen **(e, f, g, h)** on 4-16 dpi. Glomerular corpuscular necrosis and detachment from the arterioles **(i, j)** and basophilic ground-glass bodies in the cells of proximal convoluted tubules, pyknosis, and margination of the nucleus **(k, l)** in CP-LD infected hamsters on 16 dpi. The images **a, e, i, c, g, k** are 100x and **b, f, j, d, h, l** are 400x magnifications. Scale bar represents 100 µm (**b, f, j, d, h, l**) or 400 µm (**a, e, i, c, g, k**). **LD**-low dose; **HD**-high dose; **CP-LD**-cyclophosphamide treated (immunosuppressed) and low dose infected groups.

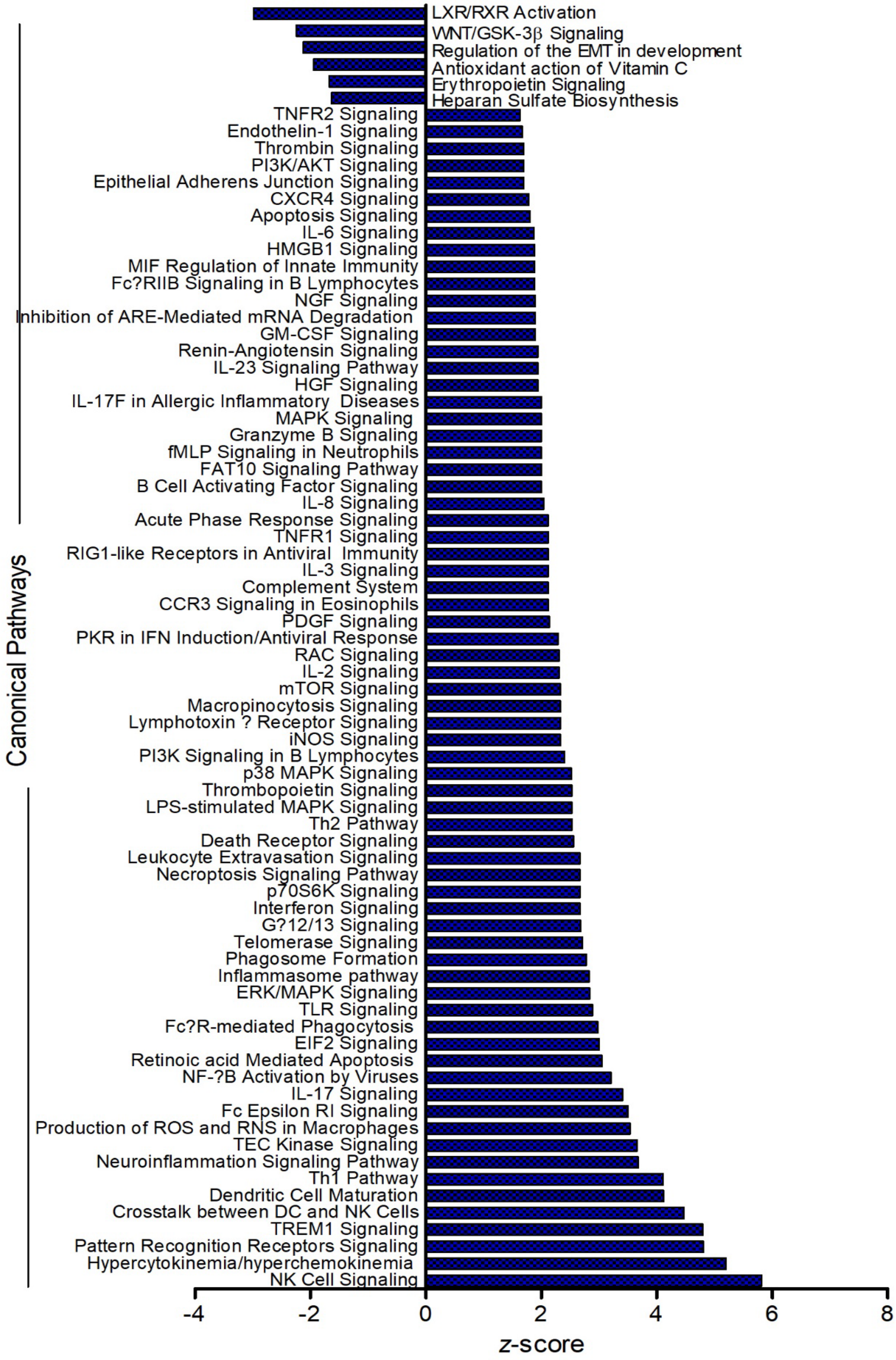
LXR/RXR Activation
WNT/GSK-3β Signaling
Regulation of the EMT in development
Antioxidant action of Vitamin C
Erythropoietin Signaling
Heparan Sulfate Biosynthesis
TNFR2 Signaling
Endothelin-1 Signaling
Thrombin Signaling
PI3K/AKT Signaling
Epithelial Adherens Junction Signaling
CXCR4 Signaling
Apoptosis Signaling
IL-6 Signaling
HMGB1 Signaling
MIF Regulation of Innate Immunity
Fc?RIIB Signaling in B Lymphocytes
NGF Signaling
Inhibition of ARE-Mediated mRNA Degradation
GM-CSF Signaling
Renin-Angiotensin Signaling
IL-23 Signaling Pathway
HGF Signaling
IL-17F in Allergic Inflammatory Diseases
MAPK Signaling
Granzyme B Signaling
fMLP Signaling in Neutrophils
FAT10 Signaling Pathway
B Cell Activating Factor Signaling
IL-8 Signaling
Acute Phase Response Signaling
TNFR1 Signaling
RIG1-like Receptors in Antiviral Immunity
IL-3 Signaling
Complement System
CCR3 Signaling in Eosinophils
PDGF Signaling
PKR in IFN Induction/Antiviral Response
RAC Signaling
IL-2 Signaling
mTOR Signaling
Macropinocytosis Signaling
Lymphotoxin ? Receptor Signaling
iNOS Signaling
PI3K Signaling in B Lymphocytes
p38 MAPK Signaling
Thrombopoietin Signaling
LPS-stimulated MAPK Signaling
Th2 Pathway
Death Receptor Signaling
Leukocyte Extravasation Signaling
Necroptosis Signaling Pathway
p70S6K Signaling
Interferon Signaling
G?12/13 Signaling
Telomerase Signaling
Phagosome Formation
Inflammasome pathway
ERK/MAPK Signaling
TLR Signaling
Fc?R-mediated Phagocytosis
EIF2 Signaling
Retinoic acid Mediated Apoptosis
NF-?B Activation by Viruses
IL-17 Signaling
Fc Epsilon RI Signaling
Production of ROS and RNS in Macrophages
TEC Kinase Signaling
Neuroinflammation Signaling Pathway
Th1 Pathway
Dendritic Cell Maturation
Crosstalk between DC and NK Cells
TREM1 Signaling
Pattern Recognition Receptors Signaling
Hypercytokinemia/hyperchemokinemia
NK Cell Signaling
Canonical Pathways
-4
-2
0
2
4
6
8
z-score

Figure S6. Top canonical pathways operative in immunocompetent hamster lungs infected with low-dose SARS-CoV-2 at 4 days post-infection. Pathways with positive z-score values are upregulated/activated, and those with negative z-score values are downregulated/suppressed.

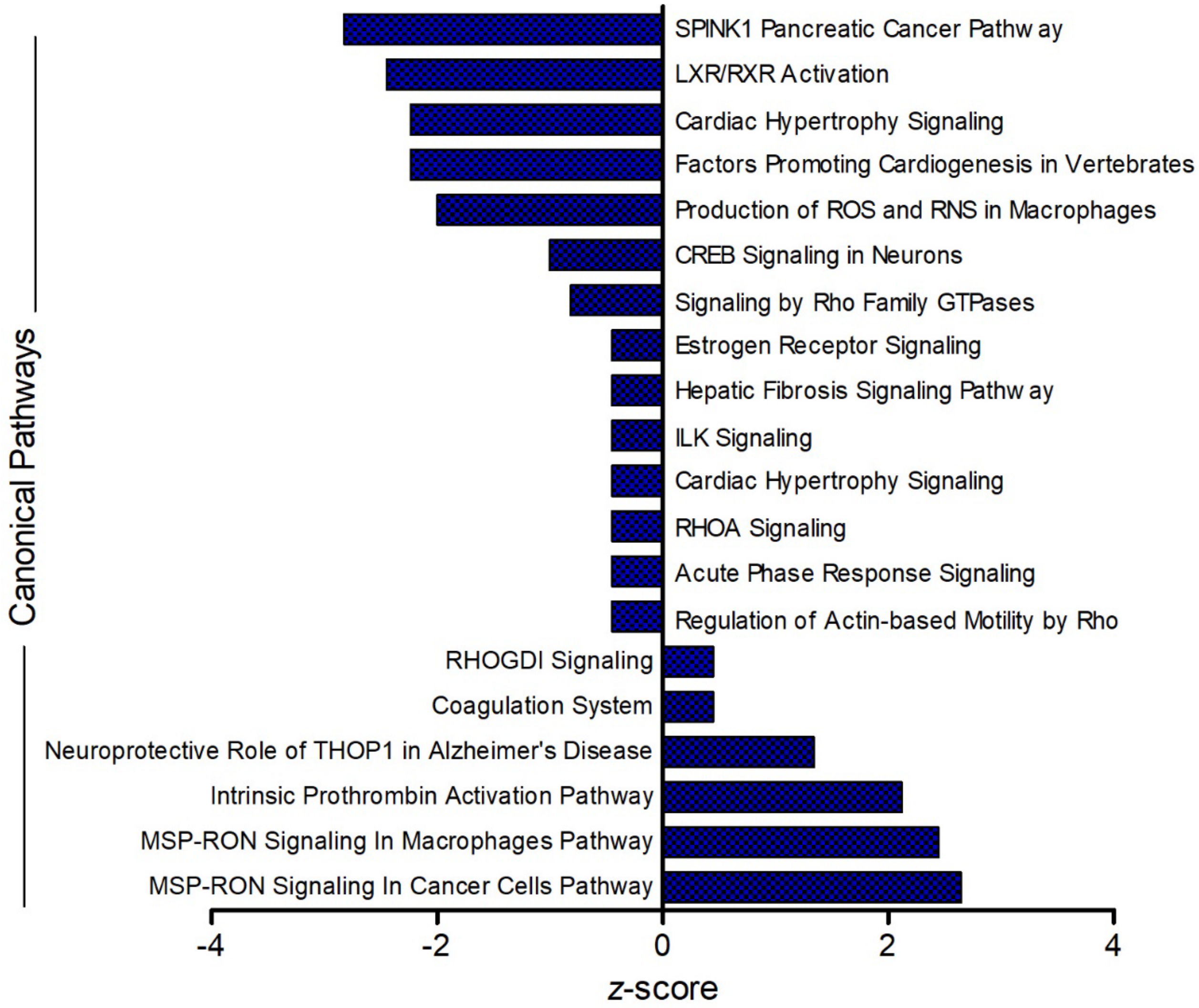

Figure S7. Top canonical pathways operative in immunocompetent hamster lungs infected with low-dose SARS-CoV-2 at 16 days post-infection. Pathways with positive z-score values are upregulated/activated, and those with negative z-score values are downregulated/suppressed.

Influenza virus activates the Toll-like receptors and cytoplasmic RIG1 in the first line of defense cells (epithelial, endothelial, macrophages, dendritic cells). Receptor activation triggers type I interferon (IFN) secretion, and the activation of further innate and adaptive immune responses.

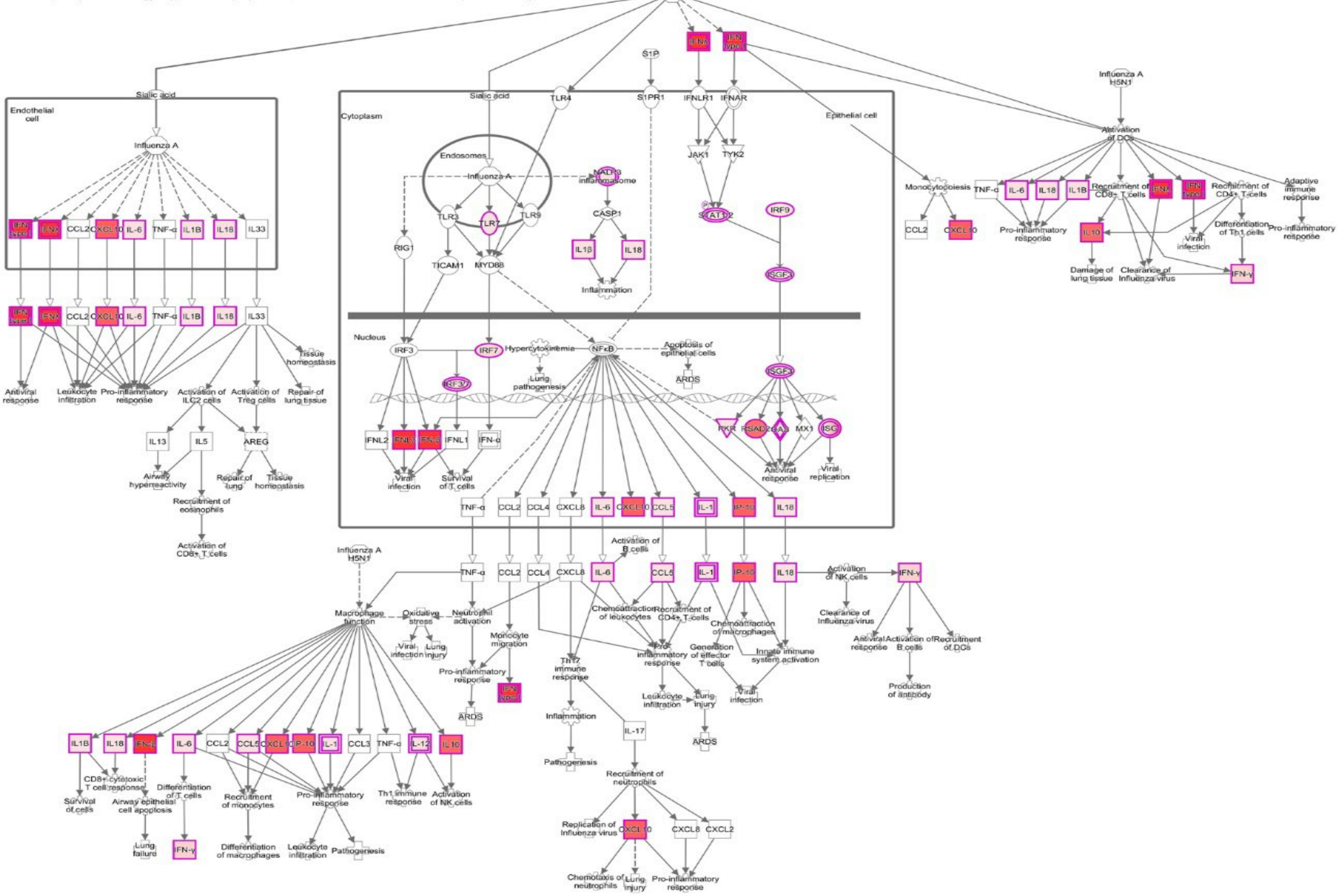

Figure S8. Canonical hypercytokinemia/hyperchemokinemia pathway map. Network genes and their interactions are shown with superimposed data from immunocompetent hamster lungs infected with low-dose SARS-CoV-2 at 4 dpi.Red color indicates upregulation, with the intensity of color proportional to the expression level (i.e., darker the color, stronger the expression).

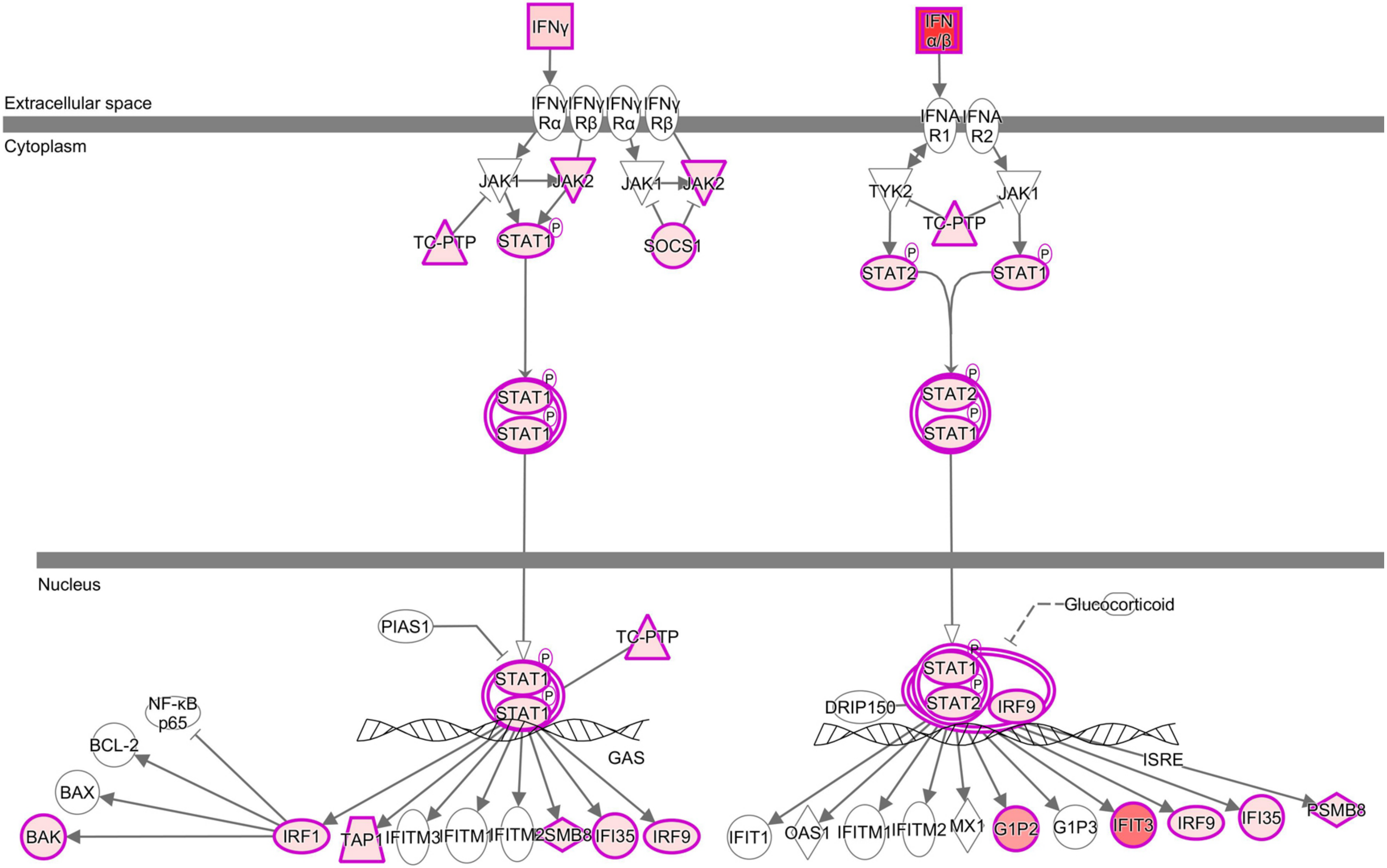
IFNγ
IFN α/β
Extracellular space
Cytoplasm
IFNγ Rα
IFNγ Rβ
IFNA R1
IFNA R2
JAK1
JAK2
TYK2
TC-PTP
STAT1
STAT2
SOCS1
P
Nucleus
PIAS1
Glucocorticoid
NF-κB p65
BCL-2
BAX
BAK
IRF1
TAP1
IFITM3
IFITM1
IFITM2
PSMB8
IFI35
IRF9
GAS
DRIP150
ISRE
IFIT1
OAS1
MX1
G1P2
G1P3
IFIT3

Figure S9. Canonical interferon signaling pathway map. Network genes and their interactions are shown with superimposed data from immunocompetent hamster lungs infected with low-dose SARS-CoV-2 at 4 dpi.Red color indicates upregulation, with the intensity of color proportional to the expression level (i.e., darker the color, stronger the expression).

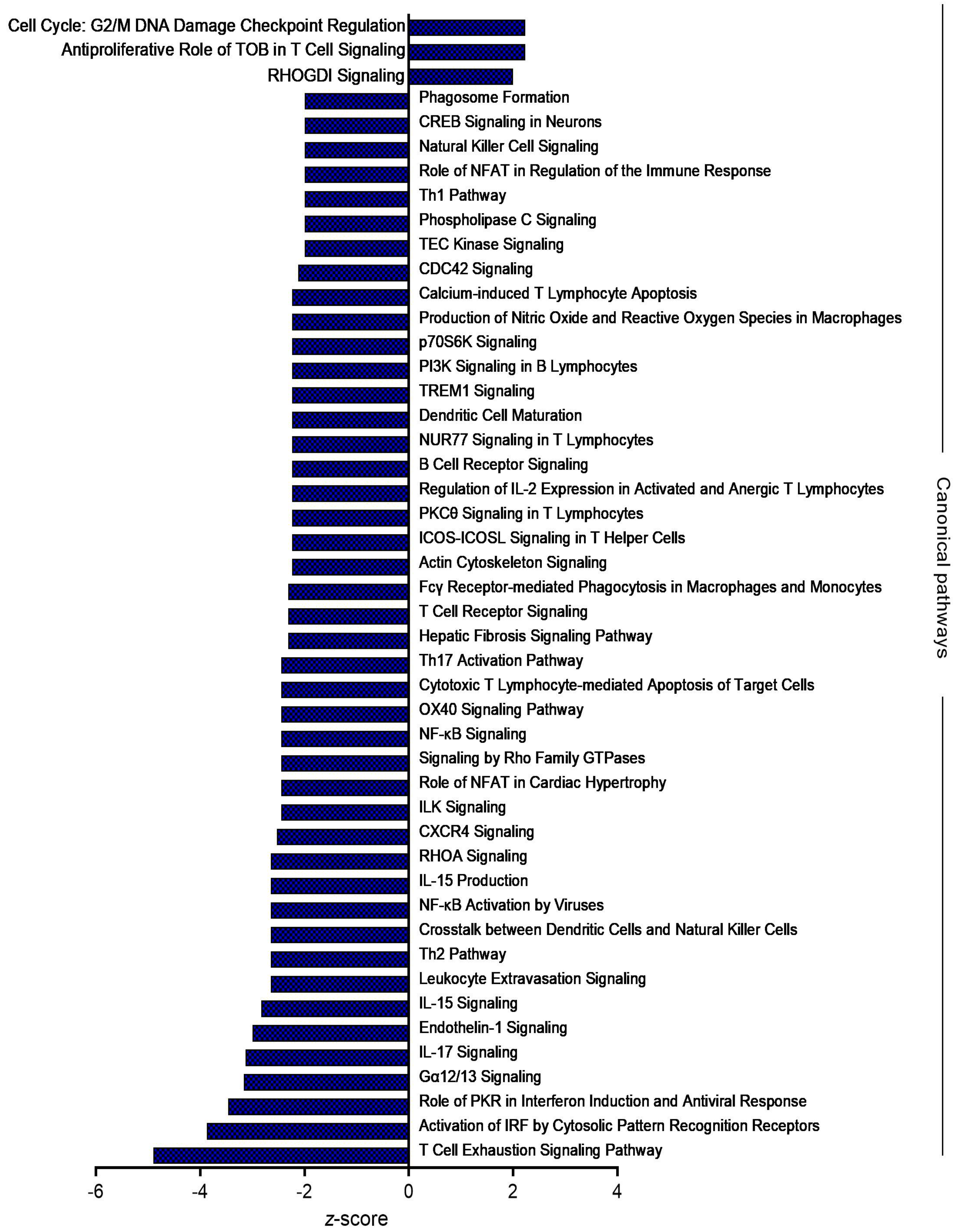
Cell Cycle: G2/M DNA Damage Checkpoint Regulation
Antiproliferative Role of TOB in T Cell Signaling
RHOGDI Signaling
Phagosome Formation
CREB Signaling in Neurons
Natural Killer Cell Signaling
Role of NFAT in Regulation of the Immune Response
Th1 Pathway
Phospholipase C Signaling
TEC Kinase Signaling
CDC42 Signaling
Calcium-induced T Lymphocyte Apoptosis
Production of Nitric Oxide and Reactive Oxygen Species in Macrophages
p70S6K Signaling
PI3K Signaling in B Lymphocytes
TREM1 Signaling
Dendritic Cell Maturation
NUR77 Signaling in T Lymphocytes
B Cell Receptor Signaling
Regulation of IL-2 Expression in Activated and Anergic T Lymphocytes
PKCθ Signaling in T Lymphocytes
ICOS-ICOSL Signaling in T Helper Cells
Actin Cytoskeleton Signaling
Fcγ Receptor-mediated Phagocytosis in Macrophages and Monocytes
T Cell Receptor Signaling
Hepatic Fibrosis Signaling Pathway
Th17 Activation Pathway
Cytotoxic T Lymphocyte-mediated Apoptosis of Target Cells
OX40 Signaling Pathway
NF-κB Signaling
Signaling by Rho Family GTPases
Role of NFAT in Cardiac Hypertrophy
ILK Signaling
CXCR4 Signaling
RHOA Signaling
IL-15 Production
NF-κB Activation by Viruses
Crosstalk between Dendritic Cells and Natural Killer Cells
Th2 Pathway
Leukocyte Extravasation Signaling
IL-15 Signaling
Endothelin-1 Signaling
IL-17 Signaling
Gα12/13 Signaling
Role of PKR in Interferon Induction and Antiviral Response
Activation of IRF by Cytosolic Pattern Recognition Receptors
T Cell Exhaustion Signaling Pathway
Canonical pathways
-6
-4
-2
0
2
4
z-score

Figure S10. Top canonical pathways operative in immunocompromised hamster lungs infected with low-dose SARS-CoV-2 at 4 days post-infection. Pathways with positive z-score values are upregulated/activated, and those with negative z-score values are downregulated/suppressed.

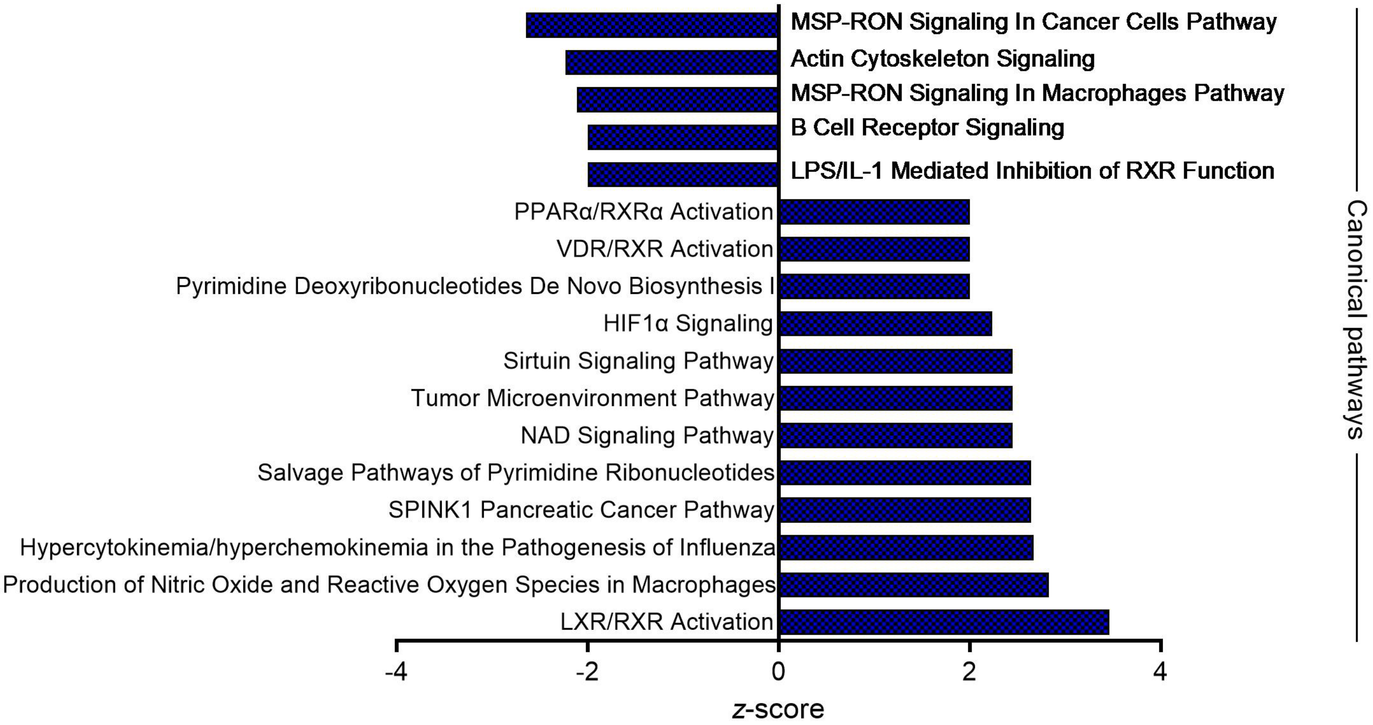

Figure S11. Top canonical pathways operative in immunocompromised hamster lungs infected with low-dose SARS-CoV-2 at 16 days post-infection. Pathways with positive z-score values are upregulated/activated and those with negative z-score values are downregulated/suppressed.

Table S1. Differentially regulated biological functions at 4dpi versus uninfected hamster lungs

Categories	***Diseases or Functions Annotation***	***p-value***	***z-score***	***# Molecules***
Antimicrobial activities of host cells	Antimicrobial response	1.74E-34	5.136	120
Cell Death and Survival	Cytotoxicity	5.04E-24	5.514	81
	Cytolysis	1.43E-24	2.991	90
Cell-mediated Immune Response	T cell development	3.02E-47	5.186	187
	Differentiation of T lymphocytes	6.43E-38	5.12	139
	T cell homeostasis	8.58E-51	5.283	195
	T cell migration	3.84E-34	5.073	111
Cell-To-Cell Signaling and Interaction	Immune response of myeloid cells	2.82E-24	5.513	77
	Response of antigen presenting cells	1.11E-26	5.295	82
	Binding of myeloid cells	3.78E-22	4.156	74
Cellular Growth, Proliferation and Movement	Stimulation of cells	1.12E-26	3.876	103
	Stimulation of lymphatic system cells	9.23E-25	3.409	62
	Stimulation of leukocytes	8.31E-25	4.241	70
	Stimulation of mononuclear leukocytes	5.29E-25	3.65	63
	Stimulation of lymphocytes	1.51E-25	3.487	61
	Stimulation of T lymphocytes	8.42E-23	3.316	53
	Recruitment of blood cells	1.8E-40	5.174	130
	Recruitment of cells	1.25E-40	4.998	137
	Recruitment of myeloid cells	7.77E-33	4.314	105
	Recruitment of leukocytes	2.36E-40	5.192	128
	Recruitment of granulocytes	1.05E-24	4.268	79
	Recruitment of phagocytes	3.33E-32	4.36	99
	Recruitment of neutrophils	6.83E-24	3.993	71
	Interaction of leukocytes	6.06E-34	6.442	131
	Binding of leukocytes	7.64E-34	6.415	130
	Interaction of mononuclear leukocytes	1.56E-23	4.975	75
	Binding of lymphocytes	9.18E-23	4.496	63
	Interaction of phagocytes	2.21E-24	4.483	77
	Activation of myeloid cells	3.02E-25	4.178	109
	Response of lymphocytes	1.6E-28	2.621	82
	Adhesion of immune cells	3.59E-32	6.276	121
	Activation of mononuclear leukocytes	3.18E-51	4.954	186
	Activation of lymphocytes	2.52E-51	4.9	181

	Activation of T lymphocytes	1.09E-45	4.555	149
	Activation of antigen presenting cells	1.74E-28	3.86	110
Inflammatory Response	Immune response of leukocytes	5.24E-41	6.073	130
	Immune response of phagocytes	1.8E-29	6.007	90
	Response of phagocytes	4.06E-31	6.004	97
	Immune response of antigen presenting cells	8.26E-26	5.226	77
	Degranulation of myeloid cells	2.78E-24	3.715	127
	Degranulation of leukocytes	1.07E-28	3.556	140
	Degranulation of phagocytes	2.44E-24	3.308	126
	Inflammatory response	1.14E-63	6.031	279
	Cell-mediated response	2.04E-31	3.697	85
	Antibody response	3.85E-23	3.625	62
	Innate immune response	1.47E-23	3.581	80
	Degranulation	3.13E-35	3.572	171
Cellular Development	Differentiation of progenitor cells	4.52E-22	3.248	129
	Proliferation of blood cells	5.84E-67	3.852	294
	Development of progenitor cells	9.63E-23	2.914	99
	Differentiation of mononuclear leukocytes	3.4E-56	6.076	239
	Development of mononuclear leukocytes	2.37E-56	5.85	239
	Differentiation of antigen presenting cells	1.49E-23	4.623	77
	Differentiation of phagocytes	3.91E-24	4.441	91
	Development of phagocytes	4.49E-22	3.759	83
	Development of hematopoietic progenitor cells	3.01E-22	2.944	94
	Proliferation of B lymphocytes	1.13E-23	4.06	94
	Proliferation of mononuclear leukocytes	2.45E-62	4.27	259
	Proliferation of immune cells	5.7E-66	4.229	274
	Proliferation of lymphocytes	3.98E-60	4.155	253
	Expansion of T lymphocytes	6.37E-32	3.308	74
	Cell proliferation of T lymphocytes	2.89E-56	3.12	217
	Expansion of mononuclear leukocytes	2.12E-32	2.918	83
	Expansion of lymphocytes	4.17E-31	2.838	80
	Expansion of leukocytes	7.59E-33	2.739	87
	Expansion of lymphoid cells	1.72E-31	2.679	81

Cellular Function and Maintenance	Cellular homeostasis	1.65E-52	5.611	425
	Function of leukocytes	1.35E-76	3.069	208
	Function of mononuclear leukocytes	5.35E-52	2.533	133
	Function of lymphocytes	1.32E-51	2.471	132
	Function of T lymphocytes	1.28E-43	2.021	109
Free Radical Scavenging	Synthesis of reactive oxygen species	4.55E-24	5.031	142
	Metabolism of reactive oxygen species	2.73E-24	4.901	148
Humoral Immune Response, Protein Synthesis	Quantity of IgG	9.45E-34	2.944	89
Immunological Disease	Hypersensitive reaction	3.59E-34	3.937	143
	Immunodeficiency	2.17E-27	-3.252	105
Organismal Survival	Organismal survival	3.17E-56	-2.157	560
	Morbidity or mortality	3.7E-59	-2.177	572

Table S2. Differentially regulated biological functions at 16dpi versus uninfected hamster lungs

Categories	Diseases or Functions Annotation	p-value	z-score	# Molecules
Cellular Movement	Cellular infiltration by myeloid cells	6.41E-06	3.241	14
	Cellular infiltration by phagocytes	2.23E-06	2.935	14
	Cellular infiltration by leukocytes	1.24E-09	2.882	22
	Cellular infiltration	2.22E-09	2.871	23
	Cellular infiltration by granulocytes	7.28E-05	2.573	10
	Infiltration by neutrophils	0.00035	2.76	8
Hematological System Development and Function	Quantity of macrophages	0.0004	2.532	9
Organismal Injury and Abnormalities	Edema	0.00045	2.747	12
Organismal Survival and Cell Death	Survival of organism	1.51E-07	-3.324	27
	Organismal death	1.41E-08	2.554	58
	Necrosis of muscle	0.000143	2.204	12
	Lung injury	1.06E-06	2.007	12
	Cell death of muscle cells	0.00045	2.033	11
Lipid Metabolism, Small Molecule Biochemistry	Fatty acid metabolism	3.54E-09	-2.253	25
	Transport of lipid	0.000118	-2.381	10
	Export of molecule	0.000937	-2.401	10
	Transport of molecule	0.00097	-2.613	32
Cardiovascular Disease and Abnormalities	Failure of heart	7.23E-07	2.63	14
	Degeneration of heart	2.16E-09	2.599	8
	Congestive heart failure	1.75E-05	2.236	8

Table S3. Differentially regulated biological functions in immunosuppressed SARS-CoV-2 infected hamster lungs at 4dpi

Categories	Diseases or Functions Annotation	p-value	z-score	# Molecules
Organismal survival	Cell survival	1.15E-17	-5.865	86
	Cell viability	2.72E-16	-6.371	81
	Cell viability of leukocytes	1.59E-24	-4.592	38
	Cell viability of lymphocytes	8.23E-20	-4.07	29
Cell Morphology	Polarization of helper T lymphocytes	4.25E-13	-2.753	11
	Polarization of lymphocytes	9.9E-15	-2.495	16
	Polarization of mononuclear leukocytes	1.33E-15	-2.645	17
	Polarization of Th1 cells	6.44E-13	-2.385	8
Cell Signaling and Molecular Transport	Flux of Ca2+	2.37E-16	-2.614	32
	Mobilization of Ca2+	2.1E-17	-2.439	34
Cell-mediated Immune Response	Differentiation of effector T lymphocytes	2.47E-11	-2.056	9
	Differentiation of T lymphocytes	5.24E-28	-2.383	50
	Proliferation of thymocytes	1.91E-14	-2.802	17
	T cell development	5.05E-42	-4.257	73
	T cell homeostasis	1.93E-44	-4.457	76
	T cell migration	4.32E-20	-2.333	36
Cell-To-Cell Signaling and Interaction	Interaction of lymphocytes	1.83E-16	-3.367	25
	Interaction of T lymphocytes	2.17E-14	-3.125	21
	Activation of lymphocytes	2.35E-42	-3.338	70
	Activation of mononuclear leukocytes	5.82E-42	-3.428	71
	Activation of natural killer cells	2.65E-12	-3.611	18
	Activation of T lymphocytes	2.23E-32	-3.199	54
Cellular Growth and Development	Maturation of blood cells	2.92E-14	-2.455	27
	Maturation of cells	2.73E-12	-2.898	34
	Proliferation of blood cells	6.93E-54	-2.429	104
	Proliferation of progenitor cells	5.63E-13	-3.388	31
	Development of B lymphocytes	1.54E-13	-2.097	20
	Proliferation of hematopoietic progenitor cells	3.24E-15	-3.216	29
	Dendropoiesis	1.9E-13	-2.81	20
	Development of antigen presenting cells	1.21E-12	-2.142	22
	Differentiation of antigen presenting cells	5.42E-16	-2.836	27
	Hematopoiesis of mononuclear leukocytes	1.87E-53	-4.181	94

	Hematopoiesis of phagocytes	1.19E-14	-2.075	25
	Leukopoiesis	3.98E-54	-4.343	101
	Development of hematopoietic progenitor cells	5.94E-17	-2.266	33
	Proliferation of immune cells	1.83E-56	-2.465	102
	Proliferation of lymphocytes	1.9E-57	-2.301	100
	Proliferation of mononuclear leukocytes	7.64E-58	-2.464	101
	Maturation of lymphocytes	3.47E-16	-3.003	21
Cellular Function and Maintenance	Cellular homeostasis	9.2E-31	-4.633	111
	Ion homeostasis of cells	2.36E-12	-2.464	40
	Lymphocyte homeostasis	5.57E-45	-4.653	78
	Function of helper T lymphocytes	4.76E-20	-2	20
Cellular Movement and Immune Cell Trafficking	Cell movement of leukocytes	1.43E-26	-2.927	71
	Cell movement of lymphocytes	7.35E-24	-2.731	48
	Cell movement of mononuclear leukocytes	4.22E-23	-2.673	51
Hematological System Development and Function	Quantity of B lymphocytes	5.47E-33	-2.303	54
	Quantity of CD4+ T-lymphocytes	4.67E-30	-2.354	41
	Quantity of lymphocytes	6.31E-49	-3.894	92
	Quantity of regulatory T lymphocytes	6.4E-13	-2.507	19
	Quantity of T lymphocytes	2.05E-38	-3.039	71
	Quantity of leukocytes	9.58E-51	-3.909	105
	Quantity of mononuclear leukocytes	3.42E-48	-3.771	93
Inflammatory Response	Cell-mediated response	1.1E-17	-2.29	28
	Inflammatory response	2.56E-18	-2.386	59
Cell Death and Survival	Apoptosis	3.45E-22	2.226	126
	Apoptosis of blood cells	7.07E-32	2.173	60
	Apoptosis of leukocytes	2.17E-31	2.224	57
	Apoptosis of lymphatic system cells	2.57E-28	3.066	49
	Apoptosis of lymphocytes	3.78E-28	2.963	47
	Apoptosis of mononuclear leukocytes	2.54E-29	2.895	49
	Cell death of lymphatic system cells	1.62E-33	2.698	57
	Cell death of lymphocytes	1.35E-34	2.549	56
	Cell death of mononuclear leukocytes	7.78E-36	2.464	58

	Morbidity or mortality	5.4E-15	5.817	107
	Organismal death	4.91E-14	5.658	104

Table S4. Differentially regulated biological functions in immunosuppressed SARS-CoV-2 infected hamster lungs at 16dpi

**Categories**	**Diseases or Functions Annotation**	**p-value**	**z-score**	**# Molecules**
Antimicrobial and Inflammatory Response	Antimicrobial response	3.65E-18	2.177	40
Cellular Movement and Cellular immunity	Cell movement	8.33E-13	2.825	118
	Migration of cells	1.66E-12	2.626	109
	Migration of mononuclear leukocytes	4.73E-10	2.138	33
	T cell migration	2.47E-09	2.223	26
	Cell movement of T lymphocytes	1.01E-07	2.119	22
	Movement of CD4+ T-lymphocytes	5.62E-07	2.295	11
Cellular Function and Maintenance	Ion homeostasis of cells	7.37E-07	2.131	35
Humoral Immune Response	Quantity of lymphoid tissue	4.37E-10	-2.022	31
	Quantity of B lymphocytes	1.56E-12	-2.696	35
	Quantity of marginal-zone B lymphocytes	1.86E-07	-2.06	12
	Quantity of IgG3	1.85E-09	-2.125	13
	Quantity of IgG2b	4.97E-08	-2.234	11
Lipid Metabolism and Molecular Transport	Transport of lipid	4.58E-12	3.482	27
	Transport of fatty acid	6.46E-08	2.762	11
	Flux of lipid	9.23E-08	2.519	15
	Transport of steroid	2.75E-07	2.199	16
	Efflux of lipid	3.47E-07	2.359	14
	Efflux of cholesterol	6.07E-07	2.003	13
	Fatty acid metabolism	9.78E-12	2.871	45
	Transport of carboxylic acid	3.18E-07	2.915	13
	Transport of molecule	1.67E-06	3.905	72
	Protein-protein	6.99E-08	-2.578	18
Organismal Injury and Abnormalities	Fibrosis	6.92E-14	-2.792	57
	Morbidity or mortality	9.64E-12	-3.901	118
	Organismal survival	4.96E-11	-3.853	115
	Thrombus formation	1.63E-09	-2.375	22

Table S5. List of primers used for hamster and SARS-CoV-2 genes in the present study

	Gene target	Name of the primer	Sequence 5'-3'	Product size
1	IL1B	Ham_IL1b_F1	AAAGCCTTGACCTGAGCTATC	110
		Ham_IL1b_R1	CTTCTCCACAGCCACAATGA	
2	TNFA	Ham_TNFa_F1	GGTTTACTCCCAGGTTCTCTTC	112
		Ham_TNFa_R1	GGACAGGAGGTTGACGTTAT	
3	IFNG	Ham_IFNg_F1	GCTATGTCTGGCTGCTACTG	99
		Ham_IFNg_R1	CCCTCCATTCACGACATCTAAG	
4	CCL2	Ham_CCL2_F1	CTGGACCAGAGTCAAGCTAAAT	110
		Ham_CCL2_R1	GGAAAGGCAGTAGTAGACACATTA	
5	MIP1A	Ham_MIP1a_F1	CCTCCTGCTGCTTCTTCTATG	118
		Ham_MIP1a_R1	TGCCGGTTTCTCTTGGTTAG	
6	IL10	Ham_IL10_F1	AGCGCTGTCATCGATTTCTC	90
		Ham_IL10_R1	CGCCTTTCTCTTGGAGCTTAT	
7	IL4	Ham_IL4_F1	GAAGAACTCCACGGAGAAAGAC	80
		Ham_IL4_R1	GGGTCACCTCATGTTGGAAATA	
8	IL6	Ham_IL6_F1	GACTTCCATCCACTTGTCTTCT	104
		Ham_IL6_R1	TTGGGAGTAGTGTCCTCTGT	
9	IL7	Ham_IL7_F1	ACACAGATGCTGGTGAACTG	97
		Ham_IL7_R1	TCTCTCTCAGTAGCCTCTTTAGG	
10	CCL5	Ham_CCL5_F1	TGCTTTGACTACCTCTCCTTTAC	109
		Ham_CCL5_R1	GGTTCCTTCGGGTGACAAA	
11	ACTN	Ham_b-actin_F1	CCCAGAGCAAGAGAGGTATTC	90
		Ham_b-actin_R1	GGTGCCAGATCTTCTCCATATC	
12	GAPDH	Ham_GAPDH_F1	AGAACGGGAAGCTTGTCATC	113
		Ham_GAPDH_R1	GCCAGTAGACTCCACAACATAC	
13	SARS-CoV-2-N	SARS-CoV-2 N_F1	GTGATGCTGCTCTTGCTTTG	97
		SARS-CoV-2 N_R1	GTGACAGTTTGGCCTTGTTG	
